# Glycoproteogenomics characterizes the CD44 splicing code associated with bladder cancer invasion

**DOI:** 10.7150/thno.67409

**Published:** 2022-03-28

**Authors:** Cristiana Gaiteiro, Janine Soares, Marta Relvas-Santos, Andreia Peixoto, Dylan Ferreira, Paula Paulo, Andreia Brandão, Elisabete Fernandes, Rita Azevedo, Carlos Palmeira, Rui Freitas, Andreia Miranda, Hugo Osório, Jesús Prieto, Luís Lima, André M. N. Silva, Lúcio Lara Santos, José Alexandre Ferreira

**Affiliations:** 1 Experimental Pathology and Therapeutics Group, IPO Porto Research Center (CI-IPOP), Portuguese Oncology Institute (IPO Porto), 4200-072 Porto, Portugal.; 2 RISE@CI-IPOP (Health Research Network), Portuguese Oncology Institute of Porto (IPO Porto), 4200-072 Porto, Portugal.; 3 Porto Comprehensive Cancer Center (P.ccc), 4200-072 Porto, Portugal.; 4 Institute of Biomedical Sciences Abel Salazar (ICBAS), University of Porto, 4050-313 Porto, Portugal.; 5 Center for Applied Medical Research (Centro de Investigación Médica Aplicada, CIMA), University of Navarra, 31008 Pamplona, Navarra, Spain.; 6 REQUIMTE-LAQV, Department of Chemistry, University of Aveiro, 3810-193 Aveiro, Portugal.; 7 Institute for Research and Innovation in Health (i3S), University of Porto, 4200-135 Porto, Portugal.; 8 Institute for Biomedical Engineering (INEB), University of Porto, 4200-135 Porto, Portugal.; 9 REQUIMTE-LAQV, Department of Chemistry and Biochemistry, Faculty of Sciences of the University of Porto, 4169-007 Porto, Portugal.; 10 Cancer Genetics Group, IPO Porto Research Center (CI-IPOP), Portuguese Oncology Institute (IPO Porto), 4200-072 Porto, Portugal.; 11 FP-I3ID, University Fernando Pessoa, 4249-004 Porto, Portugal.; 12 Laboratoire d'Etude du Métabolisme des Médicaments (LEMM), CEA, INRA, Université Paris Saclay, F-91191, Gif-sur-Yvette cedex, France.; 13 Immunology Department, Portuguese Oncology Institute (IPO Porto), 4200-072 Porto, Portugal.; 14 Faculty of Medicine of the University of Porto, 4200-319 Porto, Portugal.; 15 Ipatimup—Institute of Molecular Pathology and Immunology of the University of Porto, University of Porto, 4200-135 Porto, Portugal.; 16 GlycoMatters Biotech, 4500-162 Espinho, Portugal.; 17 Department of Surgical Oncology, Portuguese Oncology Institute (IPO Porto), 4200-072 Porto, Portugal.

**Keywords:** glycomics, proteogenomics, glycoproteogenomics, bladder cancer, CD44

## Abstract

**Rationale:** Bladder cancer (BC) management demands the introduction of novel molecular targets for precision medicine. Cell surface glycoprotein CD44 has been widely studied as a potential biomarker of BC aggressiveness and cancer stem cells. However, significant alternative splicing and multiple glycosylation generate a myriad of glycoproteoforms with potentially distinct functional roles. The lack of tools for precise molecular characterization has led to conflicting results, delaying clinical applications. Addressing these limitations, we have interrogated the transcriptome and glycoproteome of a large BC patient cohort for splicing signatures.

**Methods:** *CD44* gene and its splicing variants were assessed by Real Time-Polymerase Chain Reaction (RT-PCR) and RNAseq in tumor tissues. The co-localization of CD44 and short *O*-glycans was evaluated by proximity ligation assay (PLA), immunohistochemistry and double-immunofluorescence. An innovative glycoproteogenomics approach, integrating transcriptomics-customized datasets and glycomics for protein annotation from nanoLC-ESI-MS/MS experiments, was developed and implemented to identify CD44 variants and associated glycosignatures. The impact of CD44 silencing on proliferation and invasion of BC cell lines and glycoengineered cells was determined by BrdU ELISA and Matrigel invasion assays, respectively. Antibody phosphoarrays were used to investigate the role of CD44 and its glycoforms in the activation of relevant oncogenic signaling pathways.

**Results:** Transcriptomics analysis revealed remarkable CD44 isoforms heterogeneity in bladder cancer tissues, as well as associations between short CD44 standard splicing isoform (CD44s), invasion and poor prognosis. We further demonstrated that targeting short *O*-glycoforms such as the Tn and sialyl-Tn antigens was key to overcome the lack of cancer specificity presented by CD44. Glycoproteogenomics allowed, for the first time, the comprehensive characterization of CD44 splicing code at the protein level. The concept was applied to invasive human BC cell lines, glycoengineered cells, and tumor tissues, enabling unequivocal CD44s identification as well as associated glycoforms. Finally, we confirmed the link between CD44 and invasion in CD44s-enriched cells *in vitro* by small interfering RNA (siRNA) knockdown, supporting findings from BC tissues. The key role played by short-chain *O*-glycans in CD44-mediated invasion was also demonstrated through glycoengineered cell models.

**Conclusions:** Overall, CD44s emerged as biomarker of poor prognosis and CD44-Tn/ Sialyl-Tn (STn) as promising molecular signatures for targeted interventions. This study materializes the concept of glycoproteogenomics and provides a key vision to address the cancer splicing code at the protein level, which may now be expanded to better understand CD44 functional role in health and disease.

## Introduction

Bladder cancer (BC) remains a pressing health concern and encompasses significant mortality, especially when diagnosed at advanced stages 1. Cluster of Differentiation 44 (CD44) is a multifunctional and heavily glycosylated transmembrane protein 2 involved in cell-cell and cell-extracellular matrix adhesion 3, immune functions 4, 5, lymphocyte homing 6, 7, hematopoiesis 8, and oncogenic signaling. By interacting with several downstream effector proteins, it dictates cell migration and adhesion 9, 10, tumor invasion 11, and metastasis 12, 13. Hence, this glycoprotein has been found overexpressed in more aggressive bladder tumors 14, 15, being widely adopted as a biomarker of bladder cancer stem cells (CSC) 16, 17.

The human CD44 gene is located on chromosome 11p13 and consists of 19 exons 2, 18; however, the full-length protein has never been detected due to the occurrence of intense alternative splicing. Exons 1 to 16 encode the extracellular domain, exon 17 the transmembrane region, and exons 18 and 19 the intracellular domains 2. The first 5 exons are constitutively transcribed across all currently known isoforms, encoding hyaluronic acid 19, osteopontin 20, collagen 21, laminin 22, and fibronectin 23 binding sites. On the other hand, exons 6 to 14 are subjected to alternative splicing, generating a myriad of different variants whose functional implications are yet to be fully understood 2. The number of proteoforms generated by mRNA translation and processing is greatly amplified by *O*-GalNAc glycosylation, mostly occurring in CD44 variable regions that are rich in serine and threonine residues. Moreover, a multiplicity of different but closely related glycan structures may be found in the same protein, exponentiating molecular micro-, macro-, and meta-heterogeneity 2, 24. Still, few studies have addressed the CD44 glycocode in cancer and its functional implications for disease progression 25-27. Moreover, conflicting results have been generated concerning its role in disease and clinical value, which are directly linked to analytical limitations for unequivocal molecular characterization, mostly due to the lack of high-throughput approaches to characterize CD44 at the protein level 2. Misguiding nomenclature has also been posing a major limitation for comprehensive data mining and definitive molecular characterization, leading us to propose an uniformization (summarized in Figure S1) 2.

High-throughput proteomics constitutes the gold standard approach to tackle CD44 molecular heterogeneity; however, the success of current workflows is limited by the capacity of databases for protein annotation. The high degree of sequence similarity amongst proteoforms, many times differing by short and potentially heavily glycosylated peptide sequences, poses a significant limitation. As such, CD44 molecular characterization has been mainly inferred from transcripts analysis, supported by immunoassays based on antibodies that lack isoform specificity. In summary, a single omics cannot portrait its molecular complexity, delaying clinical applications. Herein, we hypothesize that multi-omics settings may be required to unequivocally characterize CD44 glycoproteoforms, combining transcriptomics, glycomics and glycoproteomics in glycoproteogenomics settings 28 for precise identification of CD44 signatures of clinical relevance, foreseeing the design of novel targeted therapies.

## Material and Methods

### Patients sampling and healthy human tissues

A retrospective series of 75 formalin-fixed paraffin-embedded (FFPE) bladder tumor tissues from the Portuguese Institute of Oncology of Porto (IPO-Porto) biobank were used for this study. Bladder tumors were surgically removed from 61 male and 14 female, ranging from 26 to 85 years of age (67 ± 11.7), admitted and treated at the IPO-Porto between 2000 and 2017. The tumors were classified as non-muscle invasive (≤T1, NMIBC; n = 34) and muscle-invasive (≥T2; MIBC; n = 41) bladder cancers. Eleven histologically normal urothelium tissue sections from healthy individuals were included. Additionally, a broad library of healthy tissues (liver, colon, small intestine, gallbladder, pancreas, thyroid, stomach, appendix, testicle, skin, breast, kidney, lung, mucosa-associated lymphoid tissue-MALT, and white blood cells) were also considered. Bladder tumor and urothelial sections were characterized in terms of CD44 isoforms by RT-PCR and glycoproteogenomics. Additionally, tumors and healthy tissues were screened for CD44 and altered glycosylation (Tn and STn antigens) by different immunoassays (immunohistochemistry, WB; PLA), double staining immunofluorescence with lectins and antibodies). All procedures were performed under the approval of the hospital's ethics committee (project reference: CES 86/017) after obtaining informed patient's consent, being the clinicopathological information obtained from patient's clinical records.

### TCGA Dataset

Updated clinical information for 413 TCGA Bladder Carcinoma (BLCA) cases corresponding to muscle invasive lesions of different stages (T2, T3, T4) and histopathological natures (papillary and non-papillary), including overall survival and disease-free survival information, were obtained from cBioPortal database (https://www.cbioportal.org/). The level-3 RNA-Seq data from 408 TCGA-BLCA cases was retrieved from Broad GDAC FIREHOSE (http://gdac.broadinstitute.org/) in March 2021.

### Cell lines and cell culture conditions

BC cell lines, RT4, 5637 and T24, were acquired from ATCC and cultured in RPMI 1640 GlutaMAX™ medium (Gibco, Thermo Fisher Scientific) supplemented with 10% heat-inactivated FBS (Gibco, Thermo Fisher Scientific) and 1% penicillin-streptomycin (10,000 Units/mL penicillin; 10,000 μg/mL streptomycin; Gibco, Thermo Fisher Scientific). T24 glycoengineered cell models (T24 *C1GALT1* knock-out (KO) and T24 *C1GALT1* KO*/ST6GALNAC1* knock-in (KI)) and corresponding controls (T24 *C1GALT1* control carrying a silent mutation and T24 *C1GALT1* control/*ST6GALNAC*1 mock carrying a silent mutation and a mock vector) were generated as described by Peixoto A. *et al.^29^*. Transfected cells selection was performed based on puromycin (2 μg/mL, EMD Millipore) resistance. BC cell lines and glycoengineered models were cultured at 37 °C in a 5% CO_2_ humidified atmosphere.

### CD44 transcripts identification by Real-time polymerase chain reaction

TriPure isolation reagent (Roche Diagnostics GmbH) was used to extract total RNA from BC cells, while RNA from tissues was extracted using the Absolutely RNA FFPE Kit (Agilent), according to the manufacturer's instructions. Complementary DNA (cDNA) reverse transcription and mRNA expression were performed as previously described 30. The relative expression of total *CD44* and its isoforms was determined by RT-PCR analysis using TaqMan Gene Expression Assays (total *CD44*: Hs01075864_m1; *CD44e5-v2*: Hs01075866_m1; *CD44e5-v3*: Hs01081480_m1; *CD44e5-v8*: Hs01081475_m1, *CD44e5-e15:* Hs01081473_m1; Applied Biosystems) in a 7500 Sequence Detector (Applied Biosystems). β-2-microglobulin* (B2M)* and Hypoxanthine-guanine phosphoribosyltransferase (*HPRT*) were used for normalization, also as previously described. Details on TaqMan gene expression assays can be found in Table S1. All samples were run in duplicate and relative mRNA gene expression was calculated with the 2^-ΔCt^ formula.

### Reverse Transcriptase-Polymerase Chain Reaction and Sanger Sequencing

For CD44 transcripts characterization, RNA from T24 and 5637 cells was converted into cDNA using the High-Capacity cDNA Reverse Transcription Kit (Applied Biosystems) with random primers according to the manufacturer's instructions, and specific primer for CD44. For the latter, 1 μg of total RNA was added to 4 mM dNTP Mix and 20 pmol of a CD44 specific primer (5'-CCTTATAGGACCAGAGGTTGTGTTT-3') annealing in exon 16 (NM_000610.4) in reverse orientation, and converted to cDNA in the presence of 1 μl of MultiScribe™ Reverse Transcriptase, 1μl of RNase Inhibitor and 1x RT Buffer, in a 20 μl reaction mix. Reaction took place at 37 ºC for 120 min, followed by enzyme inactivation at 85 ºC for 5 min. For amplification of CD44 transcripts, cDNA was used in two different PCR reactions with a Forward primer for exon 5 (5'-CCCCAGCAACCCTACTGATG-3') and different Reverse primers, one to exon 8 (5'-TGAATGGCTTGGGTTCCACT-3') and one to exon 16 (same used in cDNA synthesis). Briefly, 2 μl of cDNA were mixed with 400 μM dNTPs, 200 nM of each primer and 2.5 U of Takara LA Taq DNA Polymerase in a 50 μL reaction containing 1x LA PCR Buffer II (Mg^2+^ Plus) (Takara). PCR program was the following: initial denaturation at 94 ºC for 1 min; 30 cycles of annealing at 64 ºC for 1 min and extension at 68 ºC for 4 min; and final extension at 72 ºC for 10 min. Amplified products were separated in a 2% agarose gel electrophoresis and bands were excised and purified using the GFX PCR DNA and Gel Band Purification Kit (Cytiva), according to the manufacturer´s instructions. For Sanger sequencing of purified PCR products, the BigDye Terminator V3.1 chemistry and the 3500 Genetic Analyzer (Applied Biosystems) were used, following general instructions.

### CD44 transcripts identification by RNAseq

CD44 variants were identified based on a protocol described in detail by Peixoto et al. 31. Briefly, RNA was extracted from 5637 and T24 cell pellets from three independent replicates using the RNeasy Plus Mini kit (Qiagen), pooled together for each cell line, and quantified using Qubit 2.0 Fluorometer (Thermo Fisher Scientific). RNA integrity was evaluated with Agilent TapeStation (Agilent Technologies). The NEBNext Ultra RNA Library Prep Kit was used to prepare RNA sequencing library for Illumina following manufacturer's recommendations. After validation and quantification, the sequencing libraries were clustered on one lane of a flow cell, loaded on an Illumina HiSeq 4000 instrument and sequenced using a 2 × 150 Paired End configuration. The HiSeq Control Software was used to analyze the obtained results. The gene hit counts were assessed using the feature Counts from the Subread package v.1.5.2, counting only unique reads that belong to exon regions, and then used for downstream differential expression analysis. A SNP/INDEL and a gene fusion analysis were performed using the Samtools v.1.3.1 program followed by VarScan v.2.3.9 and STAR Fusion v.1.1.0, respectively. For novel CD44 transcripts discovery, Stringtie software was used to extract the transcripts expressed in each cell line. Novel transcripts were pinpointed through the comparison between the reference annotation file and resulting gft file.

### Flow cytometry

Cells were detached using Accutase™ Cell Detachment Solution (BD^TM^), fixed with 2% paraformaldehyde (PFA; Sigma-Aldrich), and incubated with rabbit anti-CD44 polyclonal antibody (ab157107, Abcam; Table S2) using a 1:100 dilution in PBS/2% FBS for 1 h at room temperature (RT). Goat anti-rabbit IgG (H + L) cross-adsorbed secondary antibody Alexa Fluor 488 (Invitrogen) was used for CD44 detection at a 1:300 dilution in PBS/2% FBS for 15 min at RT. Data analysis was performed through CXP Software in a FC500 Beckman Coulter flow cytometer. Results represent the standard deviation of three independent experiments.

### Cell models and tumor proteins extraction

Plasma membrane proteins from BC wild type and glycoengineered cell lines were extracted by subcellular fractionation using ultracentrifugation, as previously described 32. Briefly, cultured cells were detached by scrapping with fractionation buffer (20 mM HEPES buffer (pH = 7.4), 10 mM KCl, 2 mM MgCl_2_, 1 mM EDTA and 1 mM EGTA) on ice. The cell suspension was then passed through a 27G needle, left on ice for 20 min, and then centrifuged at 720 *g* for 5 min at 4 °C to remove the nuclei. Supernatants were transferred to a new tube and recentrifuged at 10,000 *g* for 5 min at 4 °C to remove mitochondria. Samples were then transferred to polycarbonate centrifuge bottles with cap assemblies and centrifuged for 1 h at 100,000 *g* at 4 °C. The pellets were recovered, resuspended in the fractionation buffer, and passed through a 25G needle before a new centrifugation for 45 min at 100,000 *g* at 4 °C. Finally, the plasma membrane-enriched fraction was resuspended in an appropriate volume of TBS with 0.3% SDS. Regarding bladder tumors, total protein was extracted from CD44-STn/Tn expressing areas excised from formalin fixed paraffin embedded tumors using the Qproteome FFPE tissue kit (Qiagen), according to the manufacturer's instructions.

### CD44 isolation and proteolytic digestion

The Pierce^TM^ Protein G Agarose (Thermo Fisher Scientific) was used to immunoprecipitate CD44 from membrane protein extracts of 5637 and T24 wild type cells and T24 glycoengineered models (100 μg or 500 μg of starting material for WB and glycoproteomics, respectively) and tumor protein extracts (500 μg of starting material). Briefly, agarose beads were blocked with 1% Bovine Serum Albumin (BSA; Sigma-Aldrich) for 1 h at 4 ºC. Prior to immunoprecipitation (IP), membrane protein extracts were cleared with blocked agarose beads for 2 h at 4 ºC, and then, incubated at 4 ºC for 2 h with 3 µg (WB) or 6 µg (glycoproteomics) of polyclonal anti-CD44 antibody (ab157107; Abcam). The protein-antibody complexes were incubated overnight with newly blocked agarose beads at 4 ºC and eluted with SDS Sample Loading Buffer (250 mM Tris-HCl pH 6.8, 8% (*w/v*), SDS, 0.2% (*w/v*) bromophenol blue, 40% (*v/v*) glycerol, 20% (*v/v*) β-mercaptoethanol). Immunoprecipitated CD44 was used for further WB and glycoproteomics analysis. CD44 IP products were resolved by electrophoresis under denaturing conditions using 4-20% gradient precast polyacrylamide gels (Bio-Rad) and bands were excised from gels. Proteins were then reduced with 10 mM 1,4-dithiothreitol (DTT; Sigma-Aldrich) for 45 min at 56 ºC, alkylated with 10 mM iodoacetamide (Sigma-Aldrich) for 30 min in the dark, desialylated with 10 U α-neuraminidase [*Clostridium perfringens* neuraminidase Type VI (Sigma-Aldrich)]^8^ for 2 h at 37 ºC and digested overnight at 37 ºC with chymotrypsin (25 µg/mL; Promega). The proteolytic digests were then analyzed by nanoLC-MS/MS.

### Glycomics

Bladder cancer cells *O*-glycome was characterized using the Reporter/Amplification method 33, as previously described by us 31, 32. Briefly, cell culture media of semi-confluent cells was supplemented with peracetylated benzyl 2-acetamido-2-deoxy-α-D-galactopyranoside (Sigma-Aldrich, St. Louis, MO, USA) to a final concentration of 150 µM. Following 24 h incubation, glycans were isolated from the conditioned media by filtration using 10 kDa centrifugal filter (Amicon Ultra-4; Merck KGaA, Darmstadt, Germany), followed by solid-phase extraction in Sep-Pak 3 cc C18 cartridges (Waters, Milford, MA, USA). The isolated Bn-*O*-glycosides were then permethylated and analyzed by MALDI-TOF-MS on a Bruker UltrafleXtreme mass spectrometer (Bruker Daltonics). Dried samples were resuspended in methanol, mixed (1:1 sample:matrix ratio) with 2,5-dihydroxybenzoic acid (DHB; 10 mg/mL in 50% methanol and 0.1% trifluoroacetic acid; Sigma-Aldrich) and spotted onto a MTP 384 polished steel target plate (Bruker Daltonics). Spectra were acquired in positive ion reflector mode, for a mass range from 540 to 2000 kDa. Then, spectra were subjected to external calibration, using the Peptide Calibration Standard II (Bruker Corporation) combined with α-cyano-4-hydroxycinnamic acid (5 mg/mL in 50% acetonitrile and 0.1% trifluoroacetic acid; Sigma-Aldrich), and internal calibration, using a mass control list constructed by us, considering previous knowledge on bladder cancer *O*-glycosylation.

### Nano-Liquid chromatography-Tandem mass spectrometry

CD44 IP digests were analyzed by nano liquid chromatography mass spectrometry (nanoLC-MS/MS), exploring an HCD-triggered CID approach. nanoLC-HCD-MS2 was carried out in a Q-Exactive Hybrid Quadrupole-Orbitrap mass spectrometer (Thermo Scientific) coupled to an Ultimate 3000 RSLCnano system (Dionex, Thermo Scientific). Briefly, chymotrypsin digested samples were pre-concentrated in an Acclaim PepMap C18 column (100 Å, 5 mm × 300 µm, i.d. 160454, Thermo Fisher Scientific). Peptide separation was performed in an analytical EASY-Spray column (C18, 100 Å, 2 µm, 75 µm × 500 mm, Thermo Fisher Scientific) with a flow rate of 0.25 µL/min, by mixing the eluent A: 0.1% aqueous formic acid (FA) and eluent B: 0.1% FA in 80% acetonitrile (ACN), with the following gradient: 2 min (2.5% B to 10% B), 50 min (10% B to 35% B), 8 min (35% B to 99% B), and 10 min (hold at 99% B). The column was equilibrated with 2.5% B for 17 min. The mass spectrometer was operated in the positive ion mode over the *m/z* range 380-1580, and spray voltage was set at 1.9 kV. Full MS settings were the following: 70k resolution (*m/z*=200), AGC target 3×10^6^, maximum injection time 100 ms. The data-dependent parameters were: minimum AGC target 7 × 10^3^, intensity threshold 6.4 × 10^4^, charge state exclusion: unassigned, 1, 8, >8, peptide match preferred, exclude isotopes on, and dynamic exclusion of 20 s. The top 10 peaks were selected for HCD fragmentation, using the following MS/MS settings: normalized collision energy (NCE) of 27%, 35k resolution (*m/z* = 200), AGC target 2 × 10^5^, maximum injection time 110 ms, isolation window 2.0 *m/z*, isolation offset 0.0 *m/z*, HCD first mass at 110 *m/z*. Mass spectrometer was controlled by Xcalibur 4.0 and Tune 2.9 software (Thermo Scientific). The samples were then run on a nanoLC system coupled to an LTQ-Orbitrap XL mass spectrometer (Thermo Scientific) to allow characterization by collision induced fragmentation (CID). Liquid chromatography was performed on an EASY-Spray C18 PepMap, 100 Å, 2 µm, 150 mm × 75 µm (Thermo Fisher Scientific) using the same gradient mentioned above. The mass spectrometer was operated in the positive ion mode over the *m/z* range 380-1580. Nanospray voltage was set at 1.9 kV and full scan nominal resolution was 60k (*m/z* = 400). CID was triggered from a precursor ion list containing the *m/z* values that presented the HexNAc oxonium ion (*m/z* 204.087 within a ± 0.01 range) in the HCD-MS/MS spectra previously acquired. The 6 most intense ions from the customized parent list were selected for CID fragmentation with NCE = 35%, and MS/MS spectra were acquired in the linear ion trap with an isolation width of 2 Da. Specific parameters were: MS maximum injection time of 500 ms; MS/MS maximum injection time of 50 ms; AGC target 1 × 10^6^ for the Orbitrap and 1 x 10^4^ for LTQ MS^n^ analysis; dynamic exclusion 45 s; charge rejection: unassigned and 1. Mass spectrometer was controlled by Xcalibur 3.1 software (Thermo Scientific).

### Bioinformatics for CD44 glycoproteoforms identification

MS data were first converted to peak lists using Proteome Discoverer version 2.5.0.400 (Thermo Scientific), and then searched against the UniProt Homo sapiens proteome (May 5 2020; 75069 entries), using the MSPepSearch and SequestHT search engines for protein identification and the Percolator algorithm v3.05.0 for statistical validation. Prior to the search, the human proteome FASTA database was edited to include CD44 sequences inferred from RNAseq characterization of 5637 and T24 cell lines (Table S2). Searches for HCD tandem spectra were performed with a tolerance of 5 ppm for precursor and 0.02 Da for fragment ions. For CID, a tolerance of 5 ppm was admitted for precursor and 0.6 Da for fragment ions. Chymotrypsin was selected as the proteolytic enzyme and up to two missed cleavages were allowed. A new customized databased composed of high confidence identifications resulting from the initial search was then constructed for definitive CD44 glycoproteoforms identification in glycoproteogenomics settings. For cell lines, carbamidomethylcysteine (+57.0215 Da) was set as a fixed modification, while methionine oxidation (+15.9949 Da), protein N-terminal formylation (+27.994 Da), N-terminal acetylation (+42.0106 Da), Asparagine deamidation (+0.9840 Da), ammonia-loss of Cysteine N-terminal (-17.0265 Da) and glutamine to pyro-glutamine modification (-17.0265 Da) were considered as variable modifications. For tumor samples, carbamidomethylcysteine (+57.0215 Da) was elected as a fixed modification and, based on previous reports concerning the analysis of FFPE tissues 34, the following oxidative modifications were also included in the variable modifications list: lysine to aminoadipic semialdehyde (-1.0316 Da), arginine to glutamic semialdehyde (-43.0534 Da), proline to pyroglutamic acid (+13.9794 Da), tryptophan to hydroxykynurenin (+19.9898 Da), tryptophan to kynurenine (+3,9949 Da), tryptophan to N-formylkynurenine (+31.9898 Da), threonine to 2-amino-3-ketobutyric Acid (-2.0156 Da), lysine methylation (+14.0156 Da), phenylalanine, Proline, Histidine and Tryptophan hydroxylation (+15.9949 Da) and Phenylalanine, Proline, Histidine and Tryptophan carbonylation (+13.9794 Da) 35. For identification of CD44 glycoproteoforms in cell lines and tumors, the following variable modifications of serine and threonine were also considered: HexNac (+203.0794 Da), NacHexHex 365,1322 Da), HexNac(2) (+406.1588 Da), HexNacNeuAc (494,1748 Da), HexNac(2)Hex (+568.2116 Da), HexNacHexNeuAc (+656,2276 Da), HexNacHexdHex 511,1901 Da), HexNacHexNeuAc(2) (947,3230 Da), HexNac(2) Hex(2)NeuAc (1021.3598 Da). The presence of sialic acids was included to contemplate the possibility of incomplete de-sialylation. Glycoproteomics data was also analyzed using ByonicTM version 2.13.2 (Protein Metrics, Cupertino, CA, USA) using default settings 36. Glycopeptide assignment was confirmed by manual spectra interpretation using the Xcalibur^TM^ software (Thermo Scientific).

### Western and lectin blotting

Protein extracts and CD44 immunoprecipitates from 5637 and T24 wild type, glycoengineered cells and bladder tumors were separated in 4-20% precast polyacrylamide gels (Bio-Rad) and transferred onto a nitrocellulose membrane (GE Healthcare Life Sciences). STn and Tn expressions were evaluated using the anti-tag-72 antibody [B72.3 + CC49] (1 μg/mL, ab199002, Abcam; Table S2) and biotinylated *Vicia Villosa* lectin (VVA lectin, 1:1000, Vector Laboratories; Table S2), respectively. CD44 expression was screened with the anti-CD44 antibody (1:5000, ab157107, Abcam; Tables S2). Proteins were blotted with the primary antibody or lectin during 1 h at RT. The peroxidase affiniPure goat anti-mouse IgG (H+L) polyclonal antibody (1:90,000, ImmunoResearch) was used as a secondary antibody for anti-tag-72 antibody detection, and the goat anti-rabbit IgG (H+L) HPR conjugate antibody (1:60,000; Thermo Fisher Scientific) was used for anti-CD44 antibody detection, both incubated for 30 min at RT. The VECTASTAIN® Elite ABC-HRP Reagent (1:10; Vector Laboratories) was used for 15 min at RT for analysis of Tn expression. Detection of B2M with the recombinant anti-B2M antibody [EP2978Y] (ab75853, Abcam; Table S2) followed by incubation with goat anti-rabbit IgG (H+L) HPR conjugate antibody (1:60,000; 30 min at RT) was performed as loading control.

### Immunohistochemistry

FFPE bladder tumors and healthy tissue sections were screened by immunohistochemistry for CD44 and Tn and STn antigens, as previously described by Peixoto, A *et al*. 31. Tn antigen expression was evaluated using the biotinylated VVA lectin (Vector Laboratories, 40 mg/mL, 1 hour at 37 ºC; Table S2) and the detection of STn and CD44 antigens were performed using the anti-tag-72 (B72.3 + CC49; Abcam, 0.5 mg/mL, overnight at 4 ºC; Table S2) and anti-CD44 (1:5000, ab157107, Abcam; Table S2) antibodies, respectively. Lack of cross-reactivity of VVA for blood group A and AB antigens was confirmed using the anti-blood group A monoclonal antibody (HE-193, Thermo Fisher, 1:5, overnight at 4 ºC; Table S2). Sialidase treatment of tissue samples prior to anti-STn probing was also performed to confirm the presence of the glycan (Sigma-Aldrich, 0.2 mg/mL, overnight at 37 ºC). CD44 and anti-tag-72 were detected using Novolink Polymer Detection System (Leica) according to manufacturer guidelines. Biotinylated VVA was detected using Streptavidin, Horseradish Peroxidase Conjugate (Thermo Fisher, ready-to-use, 30 min, RT) followed by incubation with ImmPACT® DAB Substrate, Peroxidase (Vector, 30:1000, 5 min, RT). All images were acquired on a Motic BA310E microscope (Motic) using the Motic Images Plus 3.0 software (Motic).

### Double staining immunofluorescence

A selection of FFPE tissue sections positive for Tn and CD44 were screened for both antigens through double immunofluorescence to determine colocalization of both epitopes. Briefly, FFPE tissues were deparaffined, hydrated, and exposed to antigen retrieval with EDTA 1 mM pH8. Tn antigens were detected using 40 μg/mL FITC-labeled VVA lectin for 2 h at RT. CD44 antigen detection was achieved using an unlabeled rabbit polyclonal CD44 antibody (Abcam; Table S2) at 1:250 for 1 h at RT. An Alexa Fluor 594 anti-rabbit was used for 30 min at RT in the dark as a secondary antibody. T24 wild type cells and T24 glycoengineered models were evaluated for T, ST, Tn, STn, and CD44 to detect simultaneous expression between CD44 and these *O*-glycans. Shortly, cells were fixed with 4% PFA for 15 min, and then incubated for 1 h with FITC-labeled VVA lectin (Tn, 0.02 µg/µL; Table S2), or FITC-labeled PNA lectin (T and ST after desialylation with 70 mU α-neuraminidase, 0.02 µg/µL; Table S2), or anti-tag-72 (STn, 5 µL/well; Table S2) and CD44 (1:100; Table S2). Alexa Fluor 594 anti-rabbit and Alexa Fluor 488 anti-mouse (ThermoFisher Scientific, 1:100) were used to detect CD44 and STn primary antibodies. Nuclear counterstain was performed with 4',6'-diamidino-2-phenylindole dihydrochloride (DAPI, 2.3x10^-3^ µg/µL; Thermo Scientific); for 10 min at RT in the dark. Fluorescence images were acquired on a Leica DMI6000 FFW microscope using Las X software (Leica).

### Proximity Ligation Assay

*In situ* PLA were used for simultaneous detection of CD44 and STn antigens whenever in close spatial proximity at the cell surface in tumors and healthy tissues and glycoengineered bladder cancer cells. Cells were cultured in μ‐Chamber 8-well slides (ibidi), fixed with 4% PFA for 15 min. Bladder tumors showing co-localization of CD44 and STn by immunohistochemistry, the healthy urothelium and the other human tissues described in the “Patients sampling and healthy human tissues” sections suggesting CD44 and STn co-localization were evaluated. PFA-fixed cancer cells and tissue sections first undergone antigen retrieval for 15 min with boiling citrate buffer pH=6.0 (Vector Laboratories), followed by incubation overnight at 4 °C with the conjugated primary antibodies mentioned in the immunohistochemistry section. Ligation and amplification of the PLA signal were achieved using the Duolink PLA Technology kit (Sigma-Aldrich). All slides were incubated with DAPI and mounted using Duolink Mounting Medium. Sialidase treatment prior to antibody probing was used to confirm the specificity of the PLA signals. STn and CD44 negative bladder tumors were used as negative controls. MCR-STn+ glycoengineered cell lines expressing CD44-STn 37, 38 and/or tumor tissues were used as positive controls. The images were acquired on a Leica DMI6000 FFW microscope (Leica Microsystems) using the Las X software (Leica Microsystems).

### siRNA Silencing Assay

siRNA reverse transfection was applied to silence *CD44* in 5637, T24, and T24 glycoengineered cells *in vitro*, using two different Silencer® Select siRNAs targeting CD44 (siRNAs ID: s2681 [target exon: 2] and s2682 [target exons: 1 and 2], Invitrogen). A Silencer® Select siRNA negative control (4390843, Invitrogen) was also included. Cells were detached and seeded (100,000 cells/well) in a 24-well plate before transfection with lipofectamine RNAiMAX (Invitrogen), according to the manufacturer's instructions. In brief, Silencer® Select siRNAs and lipofectamine RNAiMAX were diluted in Opti-MEM reduced serum medium (Gibco) and incubated for 5 min at RT. Subsequently, cells were incubated with siRNA-lipofectamine complexes for 72 h at 37 °C. Cells were plated in duplicates for each experiment, and the CD44 silencing was confirmed by RT-PCR using the TaqMan Gene Expression Assay Hs01075864_m1.

### Cell proliferation Assay

The proliferation of 5637, T24 cells and T24 glycoengineered cell models was evaluated in basal and silenced-CD44 conditions, using the cell proliferation ELISA BrdU Kit (Roche Diagnostics GmbH), according to the manufacturer's instructions. The immunoassay results were monitored at 450 nm using the iMARK™ microplate reader (Bio-Rad). Cell death negative controls composed of 1% Triton-X in a complete cell culture medium were used. The results are presented as the average and standard deviation of three independent assays with three replicates each.

### Invasion Assay

The invasive capacity of 5637, T24 cells and T24 glycoengineered models, in basal and CD44-silenced conditions, was assessed using Corning BioCoat Matrigel Invasion Chambers (Corning). Briefly, 5×10^4^ cells/mL were plated onto rehydrated invasion inserts according to the manufacturer's instructions and incubated at 37 °C for 24 h. After removal of non-invasive cells, membranes were washed, and invasive cells were fixed with 4% PFA for 15 min. Membranes were then mounted with VECTASHIELD mounting medium with DAPI, and invasive cells were counted in a Leica DM2000 microscope (Leica Microsystems). Three independent experiments were performed, and cells were seeded five times for each experiment. Invasion assays were normalized to cell proliferation average (Invasion Rate).

### Phospho-Kinase Antibody Array

The relative phosphorylation levels of 37 phosphorylation sites by kinases and 49 receptor tyrosine kinases (RTK) (Figures S7-8) were determined with the Human Phospho-Kinase/RTK Array kits (ARY003C and ARY001B, respectively, R&D Systems), according to manufacturer's instructions. Briefly, T24 wild type, glycoengineered cells and corresponding controls were firstly transfected with Silencer Select siRNA targeting CD44 as described above, and then lysed to extract protein from whole cells. For each cell line 300 µg and 600 µg of protein were used for Human Phospho-Kinase and RTK Array kits, respectively. The Amersham ECL Prime Western Blotting Detection Reagent (GE Healthcare Life Sciences) was used as developing reagent. Data analysis was performed through Image Lab Software (Bio-Rad) in a ChemiDoc XRS (Bio-Rad).

### Statistical Analysis

One-away and Two-way ANOVA followed by Tukey's multiple comparisons tests and Unpaired T tests were used to determine the different expression patterns of CD44 and its isoforms in BC cells and tissues, as well as in healthy tissues (RT-PCR and flow cytometry), and to test the effect of CD44 silencing in BC cells and glycoengineered cell models in functional responses (invasion, proliferation). Differences were considered significant for *p*<0.05. All experiments were performed at least in triplicates and three replicates were conducted for each independent experiment. The results are presented as the average and standard deviation of these independent assays. For the TCGA series, the Shapiro-Wilk normality test was used to determine variable normality. CD44 isoforms log2 (normalized RSEM+1) transformed expression levels were visualized as boxplots. Statistically significant differences between two groups were evaluated using the nonparametric Wilcoxon test, whereas Kruskal-Wallis tests were used for comparison of multiple groups. Spearman correlation analyses were performed to calculate correlation coefficients. Patients were separated into two groups according to the expression levels of each CD44 isoform, using the 25^th^ and 75^th^ percentiles as the cut-off point. Kaplan-Meier (K-M) survival curves were generated to compare the survival between patients with high and low expression levels. The statistical significance between the curves was determined using the log-rank test. The univariate and multivariate Cox proportional hazard regression models were performed to determine independent factors associated with prognosis. All statistical analysis were performed using R software (3.6.2), and *p*-values < 0.05 were considered statistically significant.

### Data Availability

Proteomics datasets and tables describing protein assignment glycosylation annotations have been deposited in the Proteomics Identifications Database (PRIDE; https://www.ebi.ac.uk/pride/; Project accession: PXD028307) and RNAseq was deposited in the Gene Expression Omnibus (GEO; https://www.ncbi.nlm.nih.gov/geo/) and are freely available. All other study data are included in the article and/or SI Appendix. Transcriptomics analysis of healthy tissues for CD44 isoforms were obtained from the Genotype-Tissue Expression (GTEx) Portal (https://gtexportal.org) on 03/06/21 and/or dbGaP accession number phs000424.v8.p2.

### Results and discussion

The CD44 glycoproteocode remains poorly characterized in BC and other tumors, frustrating expectations for precise clinical interventions. This is closely related to CD44 high molecular heterogeneity, resulting from intense alternative splicing, close sequence similarity between multiple proteoforms, and dense and diverse *O*-glycosylation, amplified by conflicting nomenclature. Herein, we show that multi-omics, combining transcriptomics, glycomics, and glycoproteomics in glycoproteogenomics settings, are required for precise identification of CD44 signatures of clinical relevance.

### High CD44s/st mRNA is associated with muscle invasion and worst prognosis in bladder cancer

We have started by screening by gene expression using Taqman probes the healthy urothelium (11 cases) and a broad series of bladder tumors (34 non-muscle invasive and 41 muscle-invasive bladder tumors) representative of all disease stages for total CD44 mRNA and 5 exon-exon junctions (*e5-v2*; *e5-v3*; *e5-v8*; *e5-e15*; e2(partial)-e3(partial); Table S1) frequently found in cancer models 39-43. The e5-v2, e5-v3 and e5-v8 junctions are also frequently observed in experimentally confirmed splicing isoforms such as *CD44v2-10*, *CD44v3-10*, *CD44v8-10* (Figure S1) but may also be present in many others showing variable exon combinations. On the other hand, e5-e15 and e2(partial)-e3(partial) detect shorter *CD44s/st* and *CD44sol*, respectively. For simplification and easy follow-up, we have adopted the nomenclature proposed by Azevedo, R. *et al.* 2, which reflects the nature of the transcribed exons. Notably, due to high similarity, this approach was unable to differentiate *CD44*s from the close related *CD44st* isoform containing a shorter cytoplasmic tail (Figure S1).

**Figure 1:**
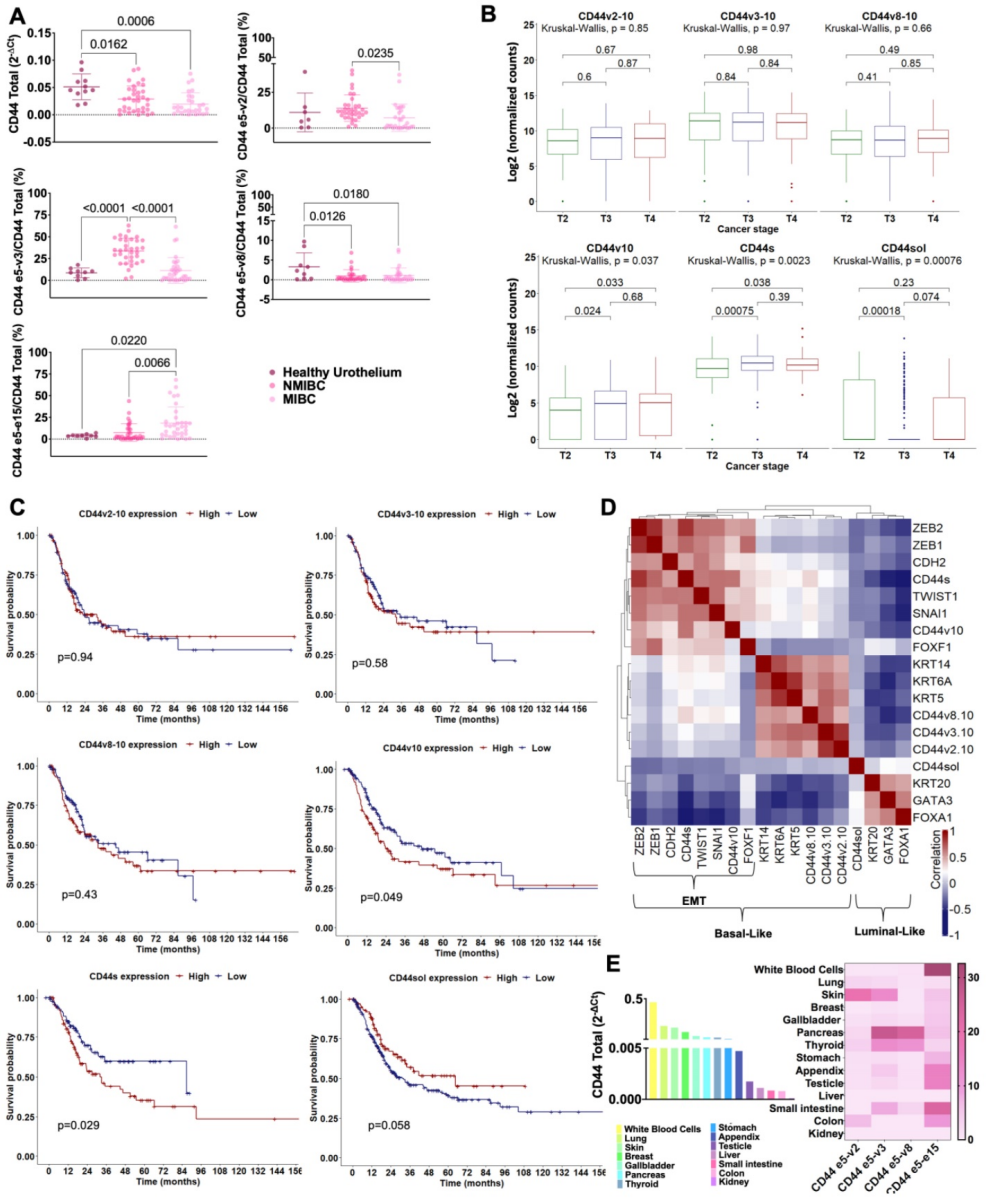
***CD44s* is increased in more aggressive bladder tumors in comparison to superficial lesions, is not present in the healthy urothelium and shows limited expression in other healthy human tumors. A)** Expression of CD44 and cancer relevant exon-exon junctions in the healthy urothelium and bladder tumors. *CD44* gene expression significantly decreased in bladder tumors in comparison to the healthy urothelium. Furthermore, the nature of the variants changed with the severity of disease, with non-muscle invasive bladder cancer (NMIBC), showing higher expression of *e5-v2* and *e5-v3* generally found in lengthier isoforms (*CD44v2-10*; *CD44v3-10*) and a significantly lower abundance of *e5-e15* characteristic of CD44s/st in comparison to muscle-invasive bladder cancer (MIBC). MIBC presented an opposite pattern, supporting enrichment for shorter CD44s isoform. The *CD44sol* isoform was also evaluated but its expression was vestigial and not linked to any tumor type, as such it was not represented in the panel A. **B)** TCGA analysis of 413 MIBC cases confirmed the association of CD44s with more aggressive late-stage disease. Briefly, *CD44s* mRNA was elevated in T3/4 tumors in comparison to T2 tumors, supporting its association with invasion, confirming the observations from our patient's dataset. **C)** Elevated *CD44v10* and *CD44s* mRNA significantly associate with worst prognosis in MIBC. The Kaplan-Meier curves highlight a clear link between the expression of shorter CD44 isoforms and worst prognosis in bladder cancer. Also, despite its low expression, patients with tumors presenting high *CD44sol* mRNA presented better prognosis. **D)** CD44s is expressed by a subgroup of basal-like tumors enriched for genes defining mesenchymal traits. In general, *CD44sol* associated with more differentiated and less aggressive luminal tumors, whereas the other forms of CD44 related with basal phenotypes, frequently less cohesive and poorly differentiated lesions. However, *CD44s and CD44v10* were characteristic of a subgroup of basal tumors enriched for *ZEB1/2, CDH2, TWIST1, SNAI1* that define mesenchymal phenotypes. Collectively, these observations link shorter CD44 isoforms to bladder cancer invasion and poor prognosis.** E)** Total *CD44* and isoforms expressions in relevant healthy cells and organs. The *CD44* gene presents a heterogeneous expression pattern in healthy tissues, and multiple isoforms may coexist in the same organ. Notably, *CD44s* was expressed by most of the studied tissues, showing significantly high mRNA levels in white blood cells. The gastrointestinal and colorectal tracts as well as the testicle also present high *CD44s*, however, with low total *CD44* levels. Collectively, this result shows that CD44s is not a cancer specific signature. The results correspond to the mean and standard deviation for three independent experiments. Triplicate measurements were conducted for each experiment. P values are presented for one-way ANOVA, nonparametric Wilcoxon, and Kruskal-Wallis tests.

In general, *CD44* mRNA was expressed more abundantly in the healthy urothelium than in bladder tumors and showed a trend to decrease with the severity of the disease (Figure 1A). Furthermore, healthy tissues and tumors presented mRNAs encoding for all targeted exon-exon junctions, supporting high microheterogeneity. This hypothesis was further reinforced by the fact that the studied isoforms only account for approximately 30% of the total *CD44* mRNA in the healthy urothelium and do not exceed 60% of *CD44* transcripts found in tumors. The existence of a more complex mixture of variable isoforms not fully captured by this approach is likely, especially for the healthy urothelium. Zooming in on the nature of the transcripts, we found that superficial tumors were enriched for variants presenting e5-v2 and e5-v3 junctions generally found in longer isoforms (namely *CD44v2-10 and/or CD44v3-10*) in comparison to muscle-invasive tumors (Figure 1A). The e5-v8 junction was elevated in cancer, irrespectively of disease stage. On the other hand, invasive tumors expressed higher percentages of shorter CD44 mRNAs lacking the variable region (*CD44s/st*). *CD44sol* mRNA was either vestigial or undetected in most samples and, therefore, has not been represented in Figure 1A**.** Collectively, these observations suggest that muscle-invasive tumors may be enriched for shorter mRNA isoforms missing variable exons in comparison to other isoforms.

We then comprehensively interrogated a larger and more homogeneous patient series comprehending over 400 muscle-invasive tumors from The Cancer Genome Atlas (TCGA) with a detailed clinical history (Figures 1B-D). We found that muscle-invasive tumors presented *CD44v2-10*, *CD44v3-10*, *CD44v8-10* and *CD44s/st* variants containing the exon-exon junctions accessed in bladder tumors by RT-PCR, as well as *CD44v10*. Even though the presence of other transcripts resulting from multiple variable exon combinations cannot be excluded, these observations reinforced the complex nature of the splice code. We started by observing that the relative abundance of these isoforms in relation to the total CD44 mRNA did not vary with the stage of disease (T2-T4; data not shown). As such, focus was on the total isoform expression foreseeing the identification of more abundant targetable signatures. *In silico* analysis revealed that late-stage invasive disease (>T2) and more aggressive non-papillary lesions 44, presented significantly higher levels of short *CD44* transcripts (*CD44sol; CD44s; CD44v10*) in comparison to T2 (Figure 1B and S2) and papillary tumors (Figure S2), respectively. Furthermore, increased *CD44*s (*p =* 0.029) and *CD44*v10 (*p =* 0.049) were significantly associated with decreased overall survival (Figure 1C). However, none of them were independent predictors of poor prognosis when adjusted to disease stage, which was a relevant variable in this context.

Finally, CD44 transcripts were evaluated in the context of transcriptome-based molecular subtypes of BC. Namely, the luminal subtype, with favorable prognosis, overexpressing *KRT20* as well as *FOXA1*, and *GATA3*, and the basal-like subtype, displaying worst prognosis, high *KRT5* and/or *KRT14* and/or *KRT6A* 45, 46. In the analysis, we also included known epithelial-to-mesenchymal transition (EMT) markers closely linked to invasion (*FOXF1*, *CDH2*, *ZEB1*, *ZEB2*, *SNAI1*, *TWIST1*). The Spearman correlation plot in Figure 1D highlights three clear clusters corresponding to these groups. Some proximity between the basal-like and EMT molecular phenotypes could also be observed, supporting the existence of a basal subgroup enriched for more invasive traits. The luminal molecular subtype was not characterized by *CD44* expression. On the other hand, high *CD44v2-10, v3-10, and v8-10* were characteristic of the basal-like group, whereas *CD44*v10 and *CD44*s were characteristic of the EMT, reinforcing the close link between shorter isoforms resulting from mRNA processing and invasive traits. Notably, in this cohort, the three subgroups presented similar survivals explained by the aggressive nature of the tumors. Nevertheless, we showed the existence of different molecular subtypes for tumors of apparently similar histology and outcome, including CD44 signatures that should be confirmed foreseeing precise cancer targeting.

Collectively, transcriptomics strongly supports the co-existence of multiple CD44 isoforms in the healthy urothelium and cancer. It also highlights the close link between the presence of shorter isoforms missing all variable exons and muscle-invasive disease, characterized by unfavorable outcomes. Nevertheless, confirmation by high-throughput proteomics is required for definitive confirmation.

### CD44s variant is expressed in healthy human tissues

Precise targeting requires significant cancer specificity. Therefore, we also evaluated a wide number of healthy tissues that exhibited variable but always detectable *CD44* mRNA expression (Figure 1E). This highlighted the complex mosaicism presented by CD44 in human organs, later confirmed by consultation of the GTEx database (Figure S3). In addition, RT-PCR (Figure 1E) and whole transcriptome analysis (Figure S3) show that, despite overexpressed in bladder cancer, CD44s may also be strongly expressed by most healthy cells, which significantly challenges its cancer specificity. Therefore, addressing CD44s post-translational modifications, namely glycosylation, poses as the next logical step towards this objective.

### BC expresses CD44 glycoforms not observed in relevant healthy cells/organs

**Figure 2:**
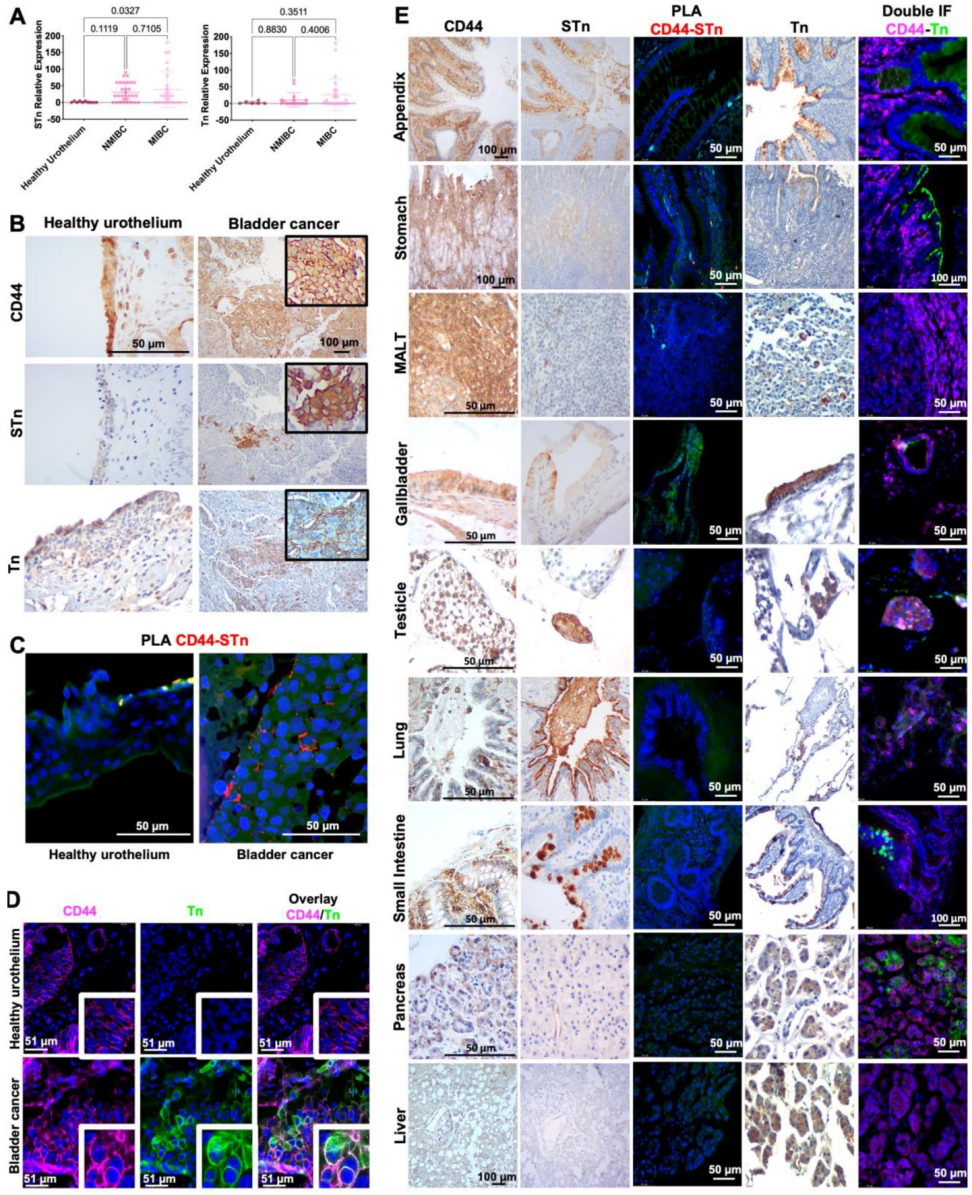
** CD44-Tn and STn glycoproteoforms present high cancer specificity. A)** Tn and STn antigens are not expressed in the healthy urothelium, are elevated in cancer, and increased in invasive tumors. The STn antigen, defined by affinity for the anti-tag-72 antibody [B72.3+CC49], is not expressed in the healthy urothelium and is elevated in cancer, being significantly overexpressed in muscle invasive bladder cancer (MIBC). The Tn antigen, based on VVA lectin immunoaffinity, is also not expressed in the healthy urothelium and is elevated in a subset of patients, specially at more advanced stages. **B)** CD44s colocalizes with Tn and STn antigens in MIBC. CD44 was diffusively expressed across the tumor section without a defined pattern. The STn antigen was observed both in superficial and invasive layers and the Tn antigen was found in scattered niches without a defined expression pattern. Immunohistochemistry also showed the co-localization of CD44 with Tn and STn positive areas in CD44s^high^ tumors, exhibiting low amounts of other isoforms (according to RT-PCR, data not shown). The healthy urothelium expressed high amounts of CD44 and the Tn antigen is present in the cytoplasm of upper stratum umbrella cells while the STn antigen was not detected. **C)** *In situ* proximity ligation assays (PLA) supports CD44s-STn glycoproteoforms in MIBC and its presence within tumor invasive fronts. *In situ* proximity ligation assay showed close spatial proximity between CD44 and STn in the same cells, strongly supporting CD44-STn glycoproteoforms in tumors. This phenotype was mostly observed in invasive fronts of CD44s^high^ tumors and was not detected in the healthy urothelium, suggesting cancer-specificity. **D)** Double staining immunofluorescence supports CD44s-Tn glycoproteoforms in bladder cancer. CD44s^high^ tumors presented niches of cells co-expressing CD44 and Tn antigen, strongly suggesting CD44-Tn glycoproteoforms. This was not observed in the healthy urothelium. **E)** Glycosylation with Tn and STn antigens provides cancer specificity to CD44. CD44 was abundantly expressed in all studied healthy tissues. STn expression was either absent or low in secretions and cells facing the lumen of the respiratory, gastrointestinal, and colorectal tracts. Immunohistochemistry suggested STn and CD44 co-localization in the stomach, appendix, small intestine, colon, gallbladder, and white blood cells in MALT. PLA did not confirm these hypotheses, suggesting cancer specificity of CD44-STn glycoproteoforms. In healthy tissues, the Tn was restricted to the cytoplasm of goblet cells in the intestinal tract, Leydig cells in testicular tissue, pancreatic acini, hepatocytes, mucinous cells of the gastric epithelium, alveolar macrophages, and gallbladder epithelium. CD44 and Tn antigen co-expression was suggested in pancreatic tissue, testicle, and gallbladder, which was not confirmed by double staining immunofluorescence.

In the past, we and other groups have demonstrated how glycosylation may increase the cancer specificity of proteins, which could be explored for precise cancer-targeting 25, 31, 32, 34, 47, 48. CD44 presents multiple potential *O*-glycosylation sites in its extracellular domain, mostly in the variable region, which could be explored towards this objective 2. We hypothesized that CD44 may reflect the profound alterations occurring in glycosylation pathways in BC, which have been directly linked to aggressive traits. As such, we have screened BC sections of different histological natures by immunohistochemistry for signs of co-localization between CD44 and the STn antigen, an immature *O*-glycan promoter of invasion 49, 50, immune escape 38, and an independent predictor of poor prognosis 51. We have also studied the Tn antigen, the shortest *O*-glycan, whose expression in BC has been linked to cancer aggressiveness in explorative studies. To avoid any possible cross-reactivity between the VVA lectin used to detect the Tn antigen and blood group A antigens, tissue sections positive for the A antigen were excluded from the analysis. According to Figures 2A-B, neither the Tn nor the STn could be observed in healthy urothelium, except for some Tn positivity in the cytoplasm of upper stratum umbrella cells. STn antigen levels were significantly elevated in tumors in comparison to the healthy urothelium, being statistically significant for invasive lesions (Figure 2A), as previously reported by us 37, 46, 51, 52. It was mostly detected at the surface of cancer cells in both superficial and invasive tumor layers, being more intense in invasive fronts in accordance with its functional role in disease progression 46, 47. The less studied Tn antigen was not significantly overexpressed in bladder tumors, except for a subgroup of patients at more advanced stages. In BC, the Tn antigen was predominantly cytoplasmic, most likely due to the detection of immature glycans in protein secretory pathways. For 47% of the cases (data not shown), membrane expression was also evident, but no pattern could be established regarding location, proximity to vessels or any other histopathological characteristics. Nevertheless, all tumors exhibiting abnormal glycosylation, also presented areas of CD44-Tn and/or STn co-expression (Figure 2B), irrespectively of their histological nature. Also, *in situ* PLA (Figure 2C) and double staining immunofluorescence (Figure 2D) for CD44 and STn and Tn antigens, respectively, revealed close spatial proximity between CD44 and the glycans in tumors but not in the healthy urothelium. Notably, positive PLA for CD44-STn was mainly observed in invasive fronts (Figure 2C) consistent with the role played individually by CD44 and STn in cancer invasion 11, 29.

We have further investigated the cancer specificity of the CD44-Tn/STn proteoforms in healthy tissues. STn expression was either absent or low in secretions and cells facing the lumen of the respiratory, gastrointestinal, and colorectal tracts (Figure 2E), in accordance with our previous reports 31, 47. Amongst STn-positive tissues, we found possible co-localization between the glycan and CD44 in the stomach, appendix, small intestine, colon, the gallbladder, and white blood cells in MALT (Figure 2E) and in peripheral blood mononuclear cells from healthy donors (data not shown). Interestingly, these organs also showed higher levels of cancer associated CD44s. However, orthogonal validation by double staining immunofluorescence and PLA did not confirm possible CD44-STn glycoproteoforms in healthy tissues, suggesting cancer specificity (Figure 2E). On the other hand, the Tn antigen was circumscribed to the cytoplasm of goblet cells at the intestinal tract, Leydig cells at testicle, pancreatic acini, hepatocytes, mucinous cells at gastric epithelium, alveolar macrophages at lung and gallbladder epithelium. Immunohistochemistry suggested some degree of overlap between Tn and CD44 in the pancreas, testicle, and gallbladder, which showed low CD44s expression (Figure 2E). However, the presence of CD44-Tn glycoproteoforms was not supported by immunofluorescence (Figure 2E). Collectively, according to our observations, short-chain *O*-glycans provide cancer-specificity to CD44 and the means for precise cancer targeting. We also identified a clear CD44s/st^high^/STn^high^ phenotype in 15% of MIBC patients, characterized by high *e5-15* transcripts (>50% in relation to total CD44; Figure 1A) and STn overexpression (>75% of the tumor area) in co-localization with CD44. These observations strongly suggest that this isoform maybe carrying altered glycosylation not observed in the healthy urothelium and mostly absent from healthy tissues.

### Glycoproteogenomics identifies multiple glycoproteoforms in BC cells

#### Transcriptomics

**Figure 3:**
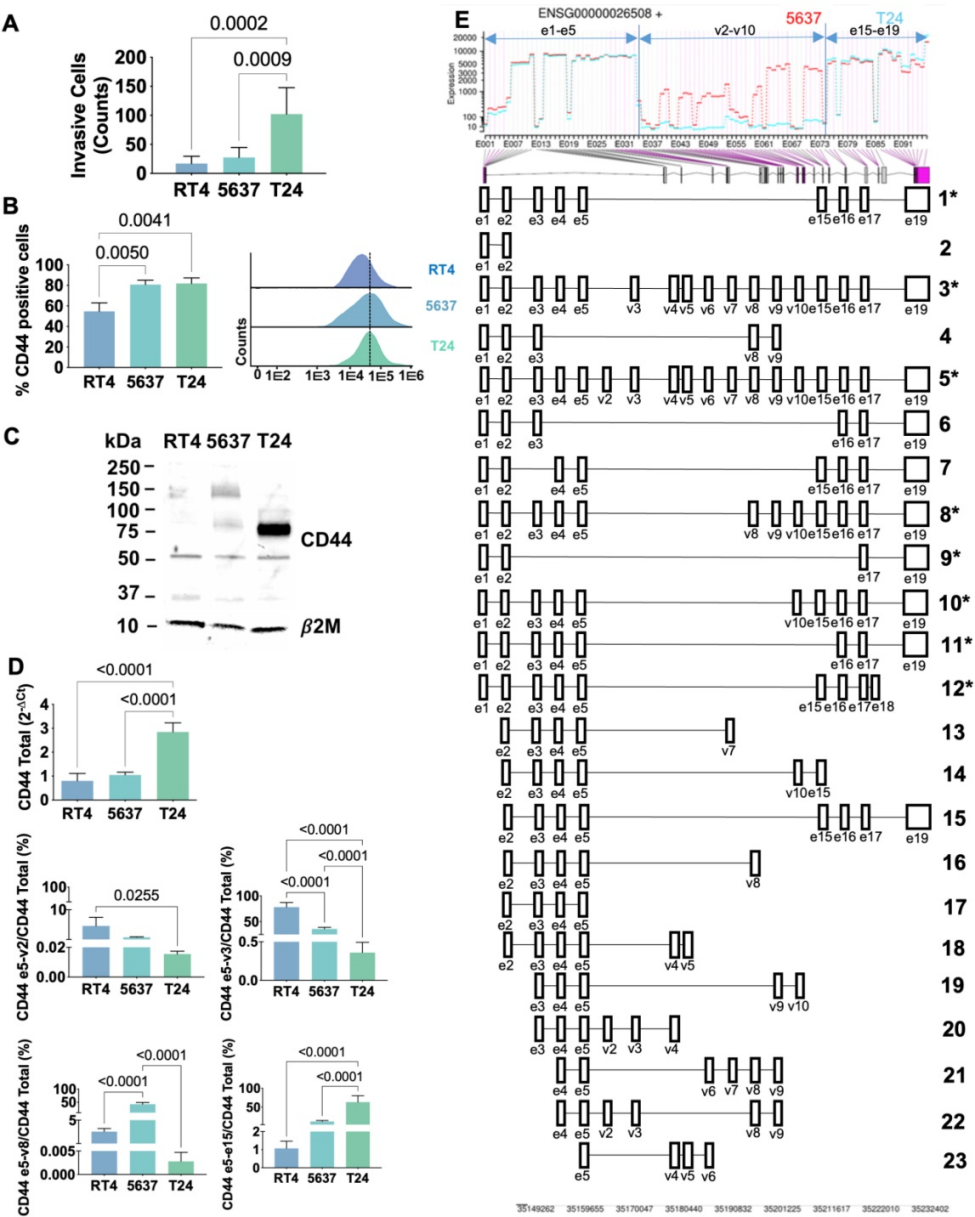
** Highly invasive T24 bladder cancer cells express high levels of CD44s. A)** Capacity to invade Matrigel *in vitro* for RT4, 5637 and T24 cells. Grades I/II cell lines (RT4 and 5637) are significantly less capable of invading Matrigel *in vitro*. **B)** CD44 expression is higher for grade II (5637) and III (T24) cells. Flow cytometry analysis showed significantly higher CD44 levels in 5637 and T24 compared to RT4 cells. **C)** Western blots show different CD44 expression patterns according to cell grade, with T24 cells showing mostly shorter proteoforms. WB confirmed the overexpression of shorter CD44 proteoforms (at approximately 75 and 50 kDa) in T24 cells, and the presence of heavier proteoforms in the other cell lines (above 150 kDa). **D)** Characterization of CD44 isoforms by RT-PCR showing isoforms shortening with cell lines aggressiveness and the marked CD44s^high^ phenotype of T24 cells. *CD44* gene expression was significantly higher in T24 in comparison to RT4 and 5637 cells, in agreement with protein analysis. RT-PCR also revealed increasing mRNA shortening with cells aggressiveness. Accordingly, RT4 showed higher *e5-v2* and *e5-v3* transcripts in comparison to the other cell lines, 5637 predominantly presented *e5-v8*, whereas T24 cells mainly expressed *e5-e15* characteristic of CD44s/st. **E)** RNAseq confirmed the marked difference between T24 and 5637 cells, the first expressing shorter CD44 mRNAs in opposition to lengthier mRNAs in 5637 cells. The top image is a DESeq2's plot for visualization of alternative splicing events. The plot shows the coverage of constitutive and variable exons for 5637 (in red) and T24 (in blue) cells. The bottom part of the image shows the gene structure and depicts differentially expressed exons (purple lines linked to the x-axis of the coverage plot). The main differences are found in the variable region spanning v2-v10, which was mostly missing in T24 cells. Predicted transcripts are shown in the bottom. Asterisks highlight those showing transcripts higher probability of generating proteins and, therefore, used for glycoprotegenomics. Legend for panel E with Uniprot and Ensemble codes: 1- P16070-12 (CD44-201); 2- E9PKC6 (CD44-222); 3- P16070-4 (CD44-206); 4- CD44-227 (no Uniprot code); 5- P16070-1 (CD44-208); 6- H0YD13 (CD44-224); 7- Q86UZ1 (CD44-207); 8- P16070-10 (CD44-209); 9- P16070-19 (CD44-203); 10- P16070-11 (CD44-210); 11- P16070-18 (CD44-205); 12- H0Y5E4 (CD44-211); 13- H0YDW7 (CD44-221); 14- H0Y2P0 (CD44-204); 15- CD44-232 (no Uniprot code); 16- H0YD17 (CD44-228); 17-H0YD90 (CD44-234); 18- CD44-213 (no Uniprot code); 19- H0YEU1 (CD44-231); 20- H0YEV3 (CD44-219); 21- H0YES0 (CD44-212). The results for A-D correspond to the mean and standard deviation for three independent experiments. Triplicate measurements were conducted for each experiment. *P* values are presented for one-way ANOVA tests.

Transcripts analysis strongly supported that the splicing code changes with cancer and varies with the different stages of the disease, which require precise identification by proteomics. Therefore, we started by choosing cell models to support the implementation of a mass-spectrometry based roadmap to address this challenge. Three well established BC cell lines reflecting distinct stages of the disease (derived from a grade I (RT4), a grade II (5637) and grade III (T24) carcinomas) were evaluated for the capability to invade Matrigel *in vitro* and screened for CD44 by real time PCR using Taqman assays. These assays were latter complemented with random and exon 16 primer specific RT-PCR followed by Sanger sequencing, RNAseq, flow cytometry, and western blotting. CD44 expression increased alongside with cell lines invasive capacity (T24>5637>RT4; Figures 3A-B), in accordance with the histopathological nature of the carcinomas from which cell lines derived. We also found that less invasive RT4 and 5637 cells expressed lower levels of CD44 in comparison to T24 cells (Figures 3B-C), being also enriched for longer CD44 variants (Figures 3C-E and S4). Interestingly, RT4 cells presented a higher number of transcripts corresponding to e5-v2 and e5-v3 junctions, whereas e5-v8 was the dominant signature in 5637 cells (Figure 3D and S4). Transcripts containing exon e15-e15 junctions characteristic of CD44s/st were much less abundant in these cell lines (>10% of total *CD44* expression; Figure 3D and S4), even though it increased in 5637 in relation to RT4 cells. On the other hand, T24 cells expressed high levels of CD44s and/or st (approximately 75% of total *CD44*) and residual amounts of other exon-exon junctions (Figures 3C-E and S4), in agreement with patient samples observations linking this variant to invasive traits. Taken together with patient samples data, these observations suggest that aggressiveness may be accompanied by a progressive loss of variable exons and, consequently, a decrease in CD44 chain length, which warrants definitive confirmation. Interestingly and in agreement with the analysis of human tissues, approximately 20-30% of *CD44* transcripts did not present any of the studied exon-exon junctions. It is probable that these cell lines may express other less abundant molecular motifs confirming the complexity of CD44 splicing code, as also suggested by exon 16 primer specific RT-PCR followed by Sanger sequencing (Figure S4) and RNAseq (Figure 3E). Namely, Figure S4 shows transcripts containing e5-v4 and e5-v10 junctions in T24 and e5-v8 in both T24 and 5637 cells, all also presenting exon 16. Alongside, RNAseq analysis also considered probable transcripts containing the following junctions: e5-v6; e5-v7; e5-v9, e3-v8, and e3-v16, to name a few (Figure 3E), which still warrant definitive confirmation by other methods. Collectively, these findings support the complex nature of the bladder cancer splicing code suggested by the analysis of tissues. Nevertheless, we considered T24 cell line as a suitable model to study the influence of CD44s/st in BC, since the other variants are practically vestigial. Moreover, based on the close similarity between RT4 and 5637, we selected 5637 and T24 cells for downstream proteomics-based studies.

In explorative studies using conventional bottom-up proteomics approaches, we concluded that the precise characterization of CD44 glycoproteoforms could only be accomplished by the customization of the databases used for protein annotation and by upfront knowledge of the cellular glycome. Through transcriptome analysis of 5637 and T24 cell lines, we discovered 38 different RNA transcripts of CD44, 18 of which have been previously described (Figures 3D-E; Table S3). Amongst these, 8 transcripts had already been experimentally validated (including CD44v2-10, CD44v3-10, CD44v8-10 and CD44s herein addressed by RT-PCR; Figure 3D) and 3 were related to non-coding RNA (Figure 3E; Table S3). The remaining 7 transcripts correspond to computationally infer short transcripts (Figure 3E; Table S3). However, half lacked open-reading frames (ENST00000526000.6 [Uniprot:H0YDW7]; ENST00000279452.10 [Uniprot:H0Y2P0]; ENST00000528455.5 [Uniprot:H0YD17]; ENST00000531873.5 [Uniprot:H0YD90]) are unlikely to be translated into proteins, while another contained a premature translation-termination codon (ENST00000425428.6 [Uniprot:Q86UZ1]). Transcript ENST00000526669.6 (Uniprot:H0YD13) encoded an incomplete extracellular domain, lacked membrane anchoring and/or intracellular region, and presented low transcript levels, suggesting reduced probability of occurrence. Finally, transcript ENST00000442151.6 (Uniprot:H0Y5E4) presents almost 100% homology with CD44st with the exception of a minor 5'truncation corresponding to the first amino acid in the protein sequence. In summary, only the computationally determined variant ENST00000442151.6 was considered for downstream validation by mass spectrometry. The remaining 20 transcripts corresponded to previously undescribed RNA sequences, probability corresponding to short non-coding transcripts, whose biological role should be investigated in the future. Collectively, transcriptomics confirmed the intense alternative splicing of CD44 in T24 cells, leading to several short transcripts encoding proteins lacking a variable extracellular region. After excluding ambiguous transcripts, a total of 9 sequences were considered plausible to generate proteins and were initially selected to construct the CD44 protein database (Table S3).

#### Glycomics

**Figure 4:**
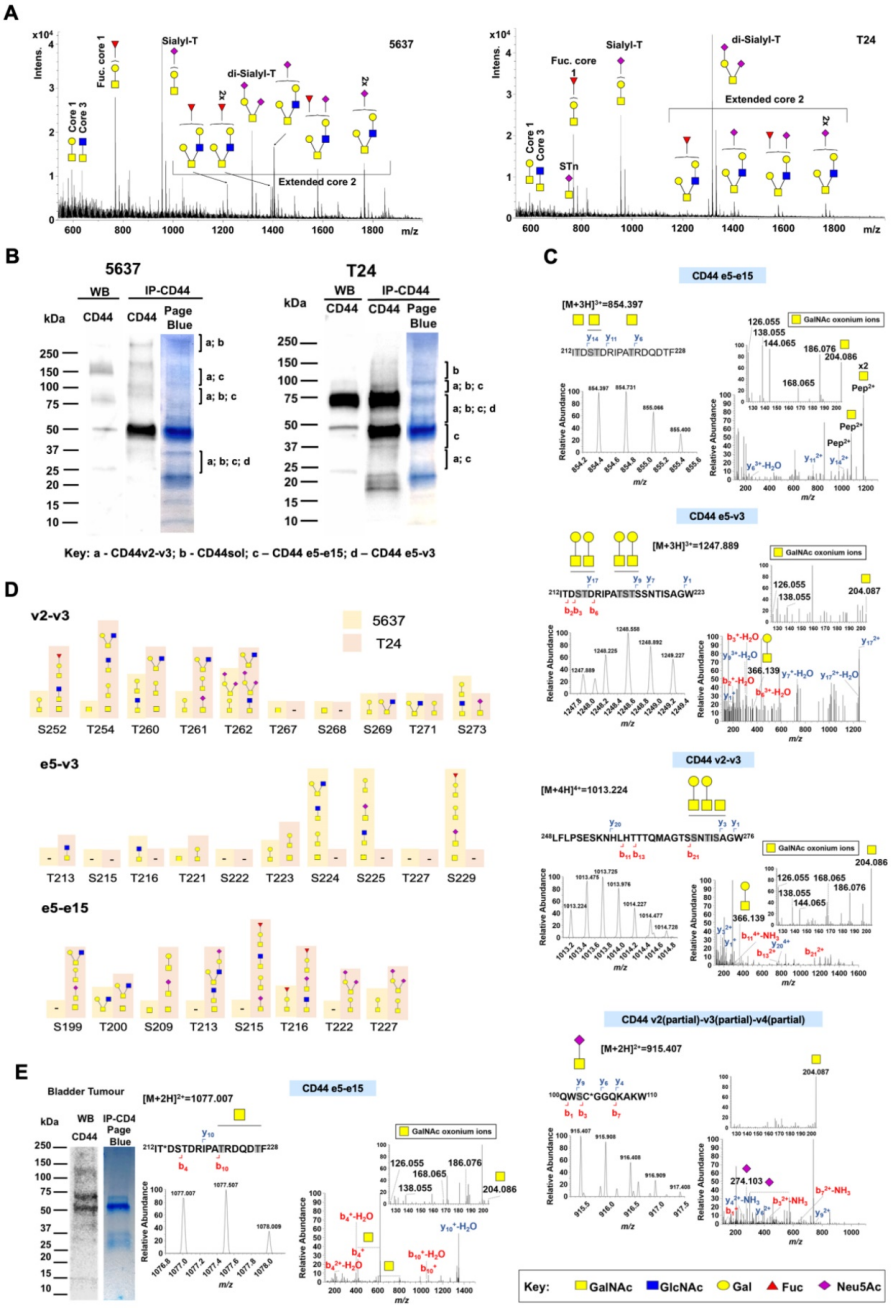
** Glycoproteogenomics, building on RNAseq-customized databases and glycomics for protein annotation, enables CD44 proteoforms identification in bladder cancer cell lines and tumors. A)** Glycomics characterization of 5637 and T24 cells showed fucosylated and sialylated T antigens as main glycospecies. MALDI-MS analysis of permethylated benzyl-GalNAc glycans revealed [M+Na]^+^ main ions for fucosylated and sialylated T antigens in both cell lines. However, 5637 predominantly expressed mono-sialylated T antigens whereas di-sialylated structures were more abundant in T24 cells. Moreover, 5637 cells presented higher abundance and diversity of glycoforms extended beyond core 2 with different degrees of sialylation and fucosylation. **B)** SDS-PAGE gels and western blots for CD44 IPs highlighting the nature of the isoforms identified by nano-LC-M/MS in glycoproteogenomics settings. Briefly, CD44 immunoprecipitated from membrane extracts was separated by gradient SDS-PAGE and bands were excised and analyzed by nanoLC-HCD/CID-MS/MS using RNAseq-customized databases and glycomics data for isoforms annotation. The nature of the isoforms translated by reporter ions for constitute-variable junctions identified in each band and cell line was highlighted. CD44 heterogeneity was evident for the two cell lines. CD44s was found to be the main isoform in T24 cells. **C)** nano-LC-MS and HCD-MS/MS spectra for reporter ions v2-v3, e5-v3, e5-e15 (CD44s/st), and e2(partial)-e3(partial) CD44sol in T24 cells. For reporter ions we show the MS isotopic envelope, the corresponding HCD product ion spectra highlighting GalNAc oxonium ions, GalNAc cross-ring fragments, other glycan fragments, and y- and b-series peptide backbone ions that support protein annotation. The predicted glycopeptide sequence, including the nature of the glycans and glycosites annotation (whenever possible) was also presented. **D)** Glycosites identified for v2-v3, e5-v3 and e5-e15 reporter glycopeptides and corresponding glycans for 5637 and T24 cells. A higher number of glycosites were identified at the e5-e15 junction for T24 in comparison to 5637 cells. On the other hand, more glycosites were identified at the v2-v3 and e5-v3 junctions in 5637 cells. **E)** nano-LC-MS and HCD-MS/MS spectra for CD44 immunoprecipitated from CD44s^high^ tumors and areas of CD44-STn co-expression. SDS-PAGE and western blots showed a pattern similar to T24 cells, characterized by bands bellow 75 kDa. An HCD product ion spectrum for an CD44s-Tn specific glycopeptide sequence is presented. Identified and possible glycosites are identified in grey. The symbol * corresponds to amino acid modifications: C - carbamidomethyl and T - 2-amino-3-ketobutyric acid.

In addition, we have characterized the 5637 and T24 cells *O*-glycome by MALDI-MS. Adding to distinct clinicopathological features, these cells exhibited slightly different *O*-glycomes in terms of glycan abundance (Figure 4A). Both cells predominantly expressed core 1 derived structures, namely fucosyl-T (*m/z* 768.38; [M+Na]^+^) and mono- (*m/z* 955.46) and di-sialylated T antigens (*m/z* 1316.64) (Figure 4A). However, in 5637, fucosylated and mono-sialylated T antigens were more abundant, whereas in T24 it was disialyl-T. The presence of several extended core 2 glycans (*m/z* 1217.60; m/z 1391.69; 1404.69; 1578.78; 1765.86) were also observed, being however more pronounced in 5637 cells. Low amounts of core 3 (*m/z* 635.32) and T (*m/z* 594.29) antigens were found in both cells as well as STn antigens (*m/z* 751.36) in T24. Overall, there were no significant differences comparing these cells *O*-glycome that could account for alteration in invasion. Namely, no significant changes were observed in terms of sialylation, fucosylation, glycosidic chains length or overexpression of immature *O*-glycans such as the Tn and STn antigens, generally associated with changes in invasion. Notably, these cells missed high levels of Tn and STn antigens highly expressed in tissue samples linked to invasion and CD44 (Figure 2), which can be explained by strong dependent on microenvironmental cues not reflected *in vitro*. Nevertheless, key information was generated to guide CD44 glycosites identification, contributing to increase protein coverage in glycoproteogenomics settings.

#### Glycoproteogenomics

For glycoproteoforms identification (Figure S5) we first immunoprecipitated CD44 from plasma membrane-enriched protein extracts isolated by differential ultracentrifugation. To ensure broad CD44 representation, we adopted an antibody targeting the cytoplasmatic tail. This avoided glycosylated regions of the protein, which may experience significant structural variations due to the dynamic nature of this post-translational modification, thus affecting antibody recognition. We first separated different proteoforms by gradient SDS-PAGE supported by western blot, excised the bands from gels (Figure 4B) and applied a proteomics workflow contemplating digestion with chymotrypsin and protein identification by nanoLC-HCD-MS/MS. This approach was complemented by CID-MS/MS triggered by the presence of the oxonium ion HexNAc (*m/z* 204.087) in the HCD-MS/MS. To render the glycosylation more homogeneous and facilitate downstream assignments by MS/MS, the glycoproteins were desialylated prior to proteolytic digestion. The western blots in Figure 4B started by highlighting multiple proteoforms across a wide range of molecular weights in BC cell lines (Figures 4C-D), as suggested by transcripts analysis. Western blots also reflected the abundance of CD44 in the cell lines, previously suggested by RT-PCR and later confirmed by flow cytometry. CD44 molecules bigger than 150 kDa predominated in 5637 cells, whereas T24 cells presented a major band at 75 kDa. Interestingly, both cell lines presented several well defined proteoforms below 37 kDa.

In explorative settings, we observed that unambiguous identification of peptides corresponding to exon-exon junctions was not possible without considering *O*-glycosylation, even when using transcriptome-customized databases. An integrative glycoproteogenomics approach was then applied, which enabled the identification of several of these junctions (detailed in Figure 4B) with high degree of confidence, based on specific diagnostic glycopeptides (Figures 4C, S6 and ST; Tables 1, S4 and S5). Using HCD fragmentation, we could identify several product ions consistent with the presence of glycosylated moieties, together with some *y-* and *b*-type peptide fragments supporting these reporter glycopeptides, even though with some ambiguity in terms of glycosites assignment (Figure 4C). The presence of GalNAc, the first sugar residue in *O*-glycans, could be further confirmed by several HexNAc cross ring fragments (*m/z* 126.055, 168.066, 186.076, and 204.087; Figures 4C-D and Figures S6-7 and 11) and, in some cases, high 144.066/138.055 oxonium ions ratios 53, 54. We also explored HexNAc-triggered CID fragmentations for further glycopeptide validation, including the characterization of extended glycan chains (Figure S6).

For certain species, CID-based fragmentation retrieved a significant number of peptide backbone fragmentations, enabling unequivocal glycosites characterization, thus in accordance with our previous reports using a similar approach 55. Notably, we found CD44 glycoproteoforms carrying both short- and elongated glycans, as well as the co-existence of different glycoforms in the same peptide sequence, even though in distinct glycosites (Figure 4C; Tables S3 and S4; Figure S6). Also, despite subjected to desialylation, some glycopeptides carrying sialic acids were still observed due to incomplete enzymatic digestion (Figures 4C and S6). Nevertheless, such observations, reinforce the tremendous molecular micro-, macro-, and meta-heterogeneity presented by CD44, previously reported for other human glycoproteins 24, portraying the relevance of glycosylation towards unequivocal molecular characterization.

SDS-PAGE-nanoLC-MS/MS analysis confirmed CD44 above 75 kDa in 5637 cells, whereas in T24 it spanned from 150 to 25 kDa (Figure 4B, Table 1), thus in accordance with the high *vs* low molecular weight splicing codes suggested by transcript analysis and western blots (Figures 3, 4B and S4). The intense band at 50 kDa in the blots may, at least partially, result from the anti-CD44 antibody used for IP, since no CD44 isoforms were identified in 5637 cells. Several CD44 glycopeptides were also found at between 37 and 25 kDa, including v2-v3 or e5-v3 exon-exon junctions. Interestingly, the corresponding variants containing all the constitutive and variable exons (CD44v2-10 and CD44v3-10, respectively) present predicted molecular weights at 80 and 75 kDa. We hypothesize that these bands may instead contain CD44 short isoforms presenting combinations of variant exons or constitutive exons which can become variable. Products of proteolytic cleavage of CD44 ectodomain by membrane-associated metalloproteases can also be found at these molecular weights and are common in cancer 56, further contributing to what we believe to be a mixture of CD44 products. The possibility of proteolysis occurring at the variable region mediated by other extracellular proteases such as metalloproteinases and ADAM has also been suggested and may also account for these findings 57, 58. In the future, efforts should be devoted to resolving these bands towards comprehensive identification.

The remaining bands also suggested that multiple isoforms could coexist in the same lane. However, the lack of MS/MS data supporting several exon-exon junctions hampers unequivocal confirmation. Nevertheless, we were able to characterize with significant detail multiple (glyco)peptides belonging to the constitutive region encoded by exons e1-e5 as well as exon e19 in both cell lines (Table 1; Figure S7). Exon-exon junctions linking the constitute region to v3 and e15 (e5-v3 and e5-e15; Table 1, Figures 4B-C and S6) were also observed in 5637 and T24. Even though we could not find e5-v2 we also successfully identified v2-v3 junctions (Figure 4C). The e5-v3 glycopeptides were found many times in association with v2-v3, v3-v4 v4-v5 and (Table 1, S4 and S5), suggesting, but not unequivocally demonstrating, multiple sequential exons. The v10-e15 junction was also frequently observed together with these structural motifs at high molecular weights (>75-250 kDa), reinforcing this hypothesis. However, we were unable to identify exon-exon junctions within the v6-v10 region. In addition, we could not identify e5-v8 junctions, even in 5637 cells that presented high e5-v8 transcripts (Figure 3D and S4). We hypothesize that the density of glycosites in this region of the protein (Figure S1) may interfere with proteolytic digestion, generating high molecular weight glycopeptides not easily identified by the adopted analytical strategy. Complementary middle-down proteomics should be considered in the future for better coverage of multiple exons, specially across densely glycosylated areas. Another relevant finding was the identification of glycopeptides for the e5-e15 junction (Table 1, S4-S5Figures 4C and S6), diagnostic of CD44s. This was associated with high CD44s coverage (>60% of the CD44s sequence; Table 1). In T24 cells, CD44s was identified in bands spanning from 150 to 37 kDa (its non-glycosylated form), including major bands at 75 and 50 kDa, in agreement with its higher abundance in these cells (Figures 3D and 4B, Table 1). The fact that CD44s and potentially other isoforms could be found spanning different molecular weights may derived from differences in glycosites occupancy and glycosidic chains, as confirmed by Table S4 and S5. Interestingly, and despite low transcription (data not shown), our glycoproteogenomics approach also identified with high coverage the CD44sol isoform (65-100%; Table 1). However, this isoform does not present nor a membrane anchoring nor a cytoplasmatic domain that enables targeting by the anti-CD44 antibody chosen for IP. Moreover, it has a predicted molecular weight of 15 kDa but has been consistently found in all bands and including at very high molecular weights. Cross-contamination is the most probable explanation, further reinforced by the fact that soluble forms of CD44 are known to bind vimentin 59 and proteoglycans 60, 61 and potentially many other yet unknown proteins. The presence of this polypeptide in most bands maybe an indicator of un-optimal SDS-PAGE 62 and/or nanoLC conditions, which should be carefully investigated and optimized in future studies.

Finally, we compared the number of glycosites identified for 5637 and T24 cells and found suggestions of isoform specific glycosylation according to the nature of the cell line. A higher number of glycosites were identified at the e5-e15 junction for T24 in comparison to 5637 cells. On the other hand, a higher number of glycosites were identified at the v2-v3 and e5-v3 junctions in 5637 cells. These findings are summarized together with the nature of the glycosidic chains in Figure 4D, but their functional meaning should carefully require evaluation in the future. We must also emphasize that the study was not designed to provide a precise identification of glycan chains, which should also be addressed in the future using dedicated protocols.

In summary, even though missing definitive confirmation regarding the composition of long isoforms presenting multiple variable exons, this strategy provide enabled the identification of CD44s diagnostic peptides. We have shown that CD44 characterization cannot be achieved by a single omics, requiring glycoproteogenomics settings. Moreover, addressing glycosylated domains is key for precise CD44 characterization. Blueprints for improving this glycoprotegenomics strategy towards more definitive CD44 splicing code characterization have been provided.

### Glycoproteogenomics identified cancer specific CD44s glycoproteoforms in bladder tumors

Transcriptomics together with different immunoassays strongly suggested but cannot present unequivocal demonstrations regarding the existence of CD44s-STn/Tn glycoproteoforms in BC. To address this limitation, our glycoproteogenomics approach was further employed to interrogate tumors for short glycoproteoforms specifically linked to BC aggressiveness. A protein pool from invasive tumors of five different patients showing high e5-e15 transcripts, CD44 western blot patterns resembling T24 cells (Figure 4D), and high STn and Tn antigens expressions was elected for this study. Immunofluorescence assays suggesting the presence of CD44-STn and Tn glycoproteoforms in the same tumor area led to consider these glycans during protein annotation. CD44-STn/Tn co-expressing areas were then excised from paraffin-embedded tumor sections after deparaffination, antigen retrieval, proteins were then extracted, and CD44 was isolated by immunoprecipitation and characterized as demonstrated for BC cell lines. Despite the significant degree of protein alterations induced by the conservation of the tumors during histological procedure, namely through multiple oxidations, we successfully identified e5-e15 glycopeptides in tumors (Figure 4D). Furthermore, we could identify the presence of short-chain cancer-associated *O*-glycans in both the CD44 constitutive region (Figure S7) as well as in CD44s reporter peptide sequences (Figure 4D). These findings provide proof of aberrant CD44s glycosylation in BC, since glycosylation with immature *O*-glycans such as the Tn and T antigens are not found in proteins of the healthy urothelium 31, 34, 52 (Figure 2) and rarely observed in healthy human tissues (Figure 2).

### CD44 is a driver of invasion

**Figure 5:**
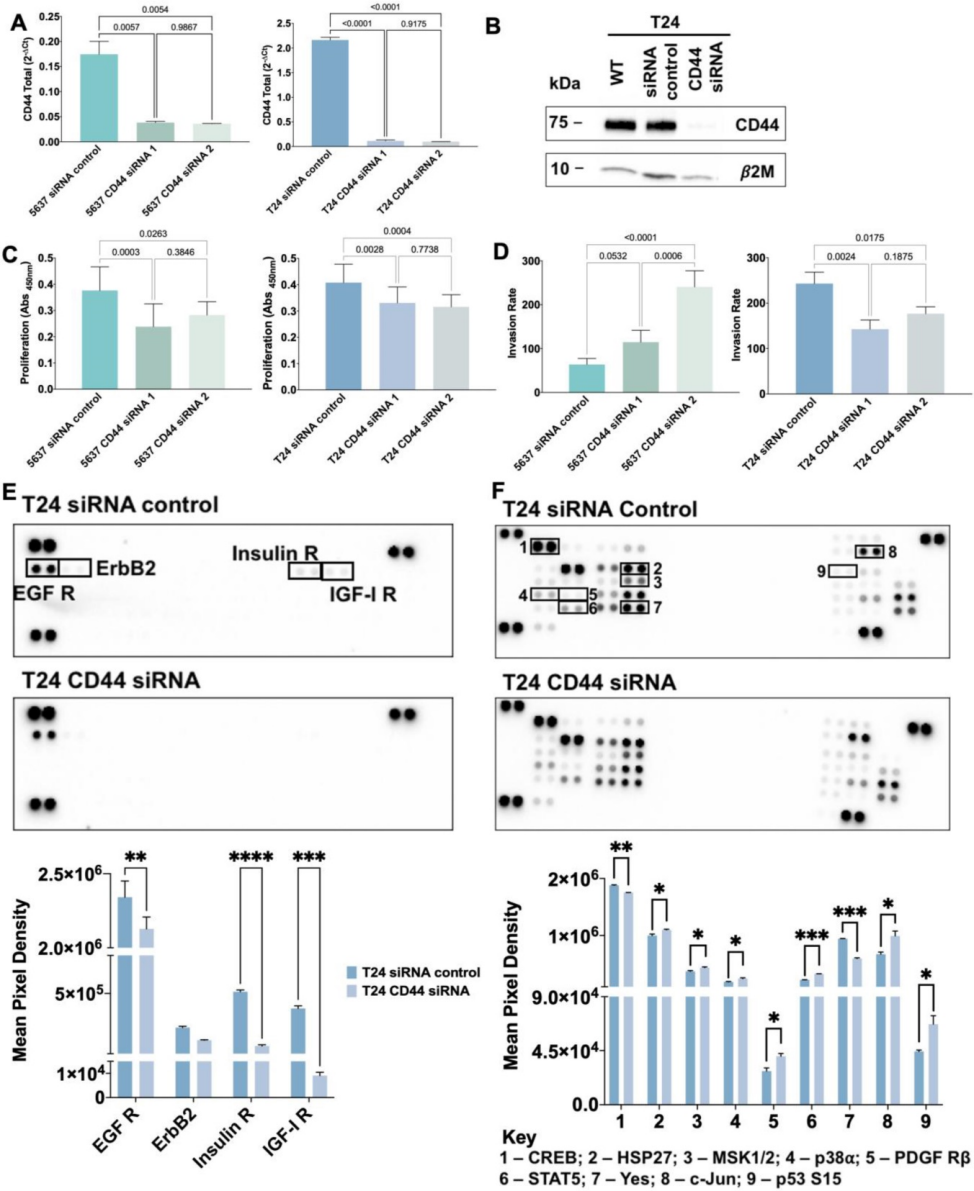
** CD44 silencing inhibits proliferation, invasion, and activation of relevant proteins in MAPK/ERK, and PI3K/AKT pathways in T24 CD44s^high^ cells. A** and **B)** CD44 siRNA promotes a massive decrease in *CD44* gene expression and complete abrogation of CD44 protein levels in 5637 and T24 cell lines. According to A, *CD44* gene expression after siRNA is decreased by approximately 80% and 96% in 5637 and T24 cells, respectively, in comparison to controls, which translated into significantly decreased protein expression (exemplified in Figure B for T24 cells). **C)** CD44 is a promoter of proliferation in 5637 and T24 cells. Proliferation was reduced by more than 50% after CD44 siRNA silencing in both cell lines compared to controls. **D)** CD44 silencing decreases the invasive capacity of CD44s^high^ T24 cells and promoted invasion in 5637 cells. The number of T24 invading cells decreased approximately 1.7-fold after CD44 knock-down comparing to controls. On the other hand, the number of cells invading matrigel *in vitro* was 1.7-fold higher for 5637 cells under the same stimuli. **E)** Analysis of the phosphorylation status of 49 RTK and **F)** Effect of CD44 silencing in phosphorylation of 37 proteins activated by different kinases. CD44s seems to be a modulator and a co-receptor of different RTKs, including EGFR, Insulin, and IGF-1 receptors, as well as transcription factors such CREB, involved in tumor proliferation and invasion. The results correspond to mean and standard deviation for three independent experiments. Triplicate measurements were conducted for each experiment. *P* values are presented for Unpaired T tests.

We then investigated the functional role played by CD44 in proliferation and invasion by silencing its expression in 5637 and T24 cells using siRNA targeting the constitutive region of the protein (80-90% CD44 decrease according to RT-PCR and WB; Figures 5A-B). This significantly reduced cell proliferation in both cell lines (Figure 5C) but impacted differently in terms of invasion, potentiating 5637 and decreasing the number of T24 invasive cells (Figure 5D). These are novel findings linking CD44 to cell proliferation in BC, supporting previous observations for breast 63 and colorectal 64 cancers, and suggesting that intense splicing of extracellular domains may play a key role in driving invasion, in agreement with observations from patient samples. Since the percentage of CD44s is significantly higher in CD44-dependent invasive cells (75% in T24 *vs* 5% in 5637; Figure 3D and S4) and significantly associated with invasion (Figure 1A-B), we also concluded that this isoform maybe used to specifically target more aggressive cancer cells subpopulations.

To disclose how CD44 could mediate proliferation and invasion, we used a human Phospho-RTK array to detect possible alterations in the phosphorylation levels of 49 different proteins (Figure S8) from T24 cells after CD44 siRNA-mediated knockdown. Epidermal growth factor receptor (EGFR), a cancer-associated member of the ErbB family, was significantly less activated in CD44 silenced T24 cells when compared to controls, which is consistent with CD44/EGFR interaction and synergic oncogenic signaling towards proliferation and invasion 65, 66. Similar behavior was observed for the highly homologous insulin (IR) and insulin-like growth factor 1 (IGF-1R) receptors 67 after CD44 silencing (Figure 5E). Interestingly, increased IGF-1R phosphorylation in CD44 expressing cells has been linked to concomitant PI3K/AKT/mTOR pathway activation, EMT molecular traits and stem cells maintenance in solid tumors 68. These observations support that CD44-mediated IR and IGF-1R activation, including downstream AKT and MAPK signaling transduction governing proliferation and invasion 69, 70 may also occur in BC. Altogether, CD44s, the most prevalent variant in T24 cells, seems to promote the activity of relevant RTK, culminating in biological cues that drive cancer cell proliferation and invasion. However, the exact mechanism by which CD44 triggers RTK activation remains poorly understood and should be addressed in future studies.

We further investigated how CD44 silencing would influence the activation by phosphorylation of several phospho-kinases and related downstream targets, using a human Phospho-Kinase array (Figure S9). As shown in Figure 5F, the activating phosphorylation of the transcription factor CREB as well as Src family non-RTK Yes was significantly decreased in T24 cells upon CD44 knock-down. CREB has been previously reported as a downstream target of CD44, mediating tumor proliferation, progression, and invasion in several tumor models 71, 72. CD44 knock-down also resulted in HSP27, MSK1/2, p38α, PDGFRβ, c-Jun, STAT5a/b, Yes and p53 (S15) phospho-kinase activation. In agreement with these observations, several studies have described CD44 as a negative regulator of PDGFRβ signaling by mediating the recruitment of a tyrosine phosphatase to PDGFR and by destabilizing PDGFR heteroreceptor complexes, ultimately modulating growth factor signaling and proliferation 73, 74. Moreover, CD44 has been previously described as a negative regulator of p53 mediated responses, allowing cells to resist programmed death and senescence as well as respond to proliferative signals 75, 76. The regulatory effect of CD44 on HSP27, MSK1/2, STAT5a/b and Yes are being described for the first time and warrant deeper investigation. Notably, Yes is the most widely expressed member of the Src kinase family and has a known regulatory role in growth factor signaling, cell proliferation, and invasion in different cancer models 77, 78. Our observations support that CD44 may act as a Yes regulator in BC, which also merits future validation. On the other hand, CD44 has been described as a positive regulator of p38α 79 and c-Jun 80 in other tumor models, which potentially conflicts with our current observations and suggests cell-dependent responses that may be governed by the nature of CD44 isoforms. Collectively, our observations emphasize that we are still far from fully understanding CD44 regulation of oncogenic pathways, which may now be comprehensively addressed in the context of its isoforms. Nevertheless, we provide evidence that CD44 acts as an oncogenic modulator of phospho-kinases involved in several signaling pathways that support proliferation and invasion, including ERK/MAPK, PI3K/AKT signaling pathways. The dominant nature of CD44s in relation to other isoforms in T24 cells together with observations in patient samples suggest that it may play a functional role in bladder cancer aggressiveness, including the modulation of the above-described molecular events. However, this remains still a hypothesis which warrants confirmation through specific silencing of this isoform in a close future.

### CD44 glycosylation modulates proliferation and invasion

**Figure 6:**
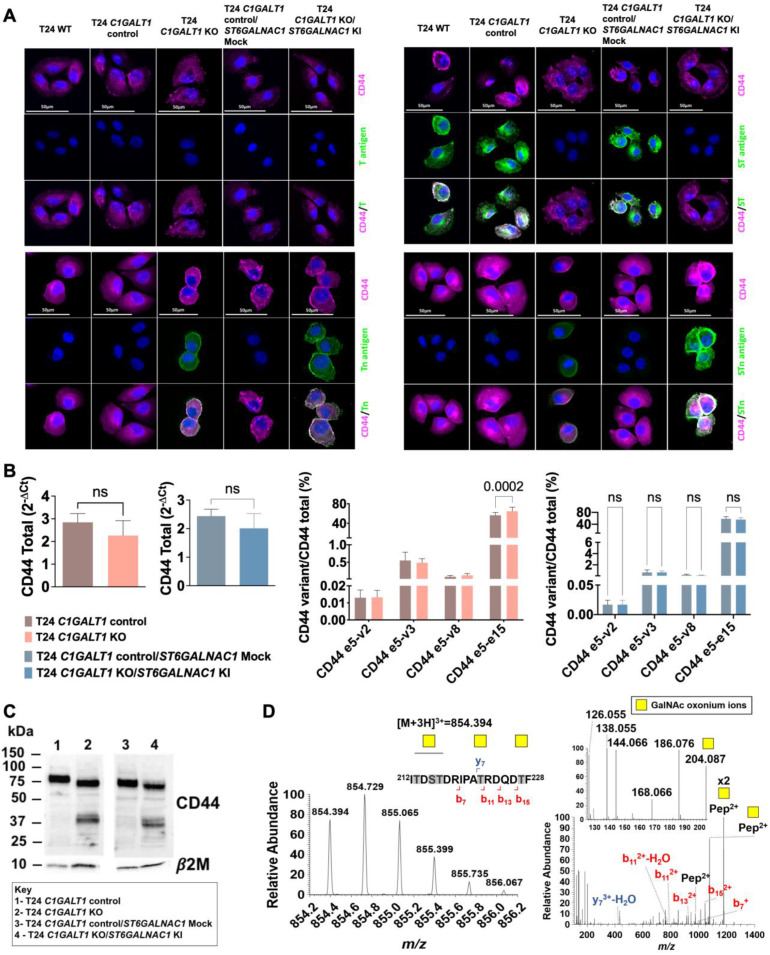
** Glycoengineered T24 cells express CD44s-Tn/STn glycoproteoforms in mimicry of human tumors. A)** Immunofluorescence studies on glycoengineered T24 bladder cancer cells demonstrated the overexpression of immature short-chain *O*-glycans together with CD44 at the cell surface of the cells. Immunoassays show high levels of sialylated T antigens and no Tn and STn antigens in wild type cells. T24 *C1GALT1* KO lacked extended glycosylation translated by the presence of sialylated T antigens and presented high Tn levels. *ST6GALNAC1* KI induced an overexpression of STn. These glycans were co-localized with CD44 at the cells surface. **B)** *CD44* gene expression and the nature of the variants do not change with alterations in glycosylation induced by *C1GALT1* KO and *ST6GALNAC1* KI, except for *CD44s*, which increased in Tn-expressing cells. **C)** Western blots show a different CD44 expression pattern for T24 glycoengineered cells in comparison to controls. The main molecular band of CD44 at 75kDa shows a slight decrease, possibly due to glycans complexity reduction. A new band at 37kDa emerged, suggesting that glycans truncation may impact on CD44 molecular weight. **D)** MS and HCD-MS/MS for a CD44s specific glycopeptide (correspoponding to the e5-e15 junction) from T24 *C1GALT1* KO cells evidencing short-chain *O*-glycosylation. The product ion spectrum shows a typical GalNAc fragmentation pattern, characterized by the presence of an evident ion at *m/z* 144.066 and a high 144.066/138.055 oxonium ions ratio, as well as the presence of the others HexNAc oxonium ions (*m/z* 126.055,168.066, 186.076 and 204.087). The spectrum also shows major fragment glycopeptides with GalNAc losses. The results correspond to the mean and standard deviation for three independent experiments. Triplicate measurements were conducted for each experiment. *P* values are presented for two-way ANOVA and Unpaired T tests.

To assess the functional impact of altered CD44 glycosylation, we explored glycoengineered T24 cells overexpressing Tn (T24 *C1GALT1* KO) and STn antigens (T24 *C1GALT1* KO/*ST6GALNAC1* KI in mimicry of aggressive bladder tumors. Tn antigen expression was accomplished by complete abrogation of *O*-glycans extension by *C1GALT1* KO, as translated by a complete loss of sialylated T antigens (Figure 6A). Alongside the Tn antigen, these cells also expressed low amounts of STn, denoting low levels of sialylation in comparison to wild type cells. Then, *ST6GALNAC1* KI was introduced to promote Tn sialylation, generating a cell line expressing high STn levels and, to less extent, Tn antigens (Figure 6A). According to immunofluorescence, all models co-expressed CD44 together with altered glycosylation at the cell surface (Figure 6A). Furthermore, changes in glycosylation did not alter *CD44* gene expression or the nature of the variants, except for CD44s, which was found increased in cells overexpressing Tn but not for cells overexpressing STn (Figure 6B). Western blots supported little changes in CD44 levels in glycoengineered models, as suggested by gene expression. However, the blots for glycoengineered cell models showed a slight decrease in the main molecular band at 75 kDa, most likely from changes in glycans complexity (Figure 6C). This hypothesis was later confirmed by CD44 immunoprecipitation assays (Figure S10) and MS/MS analysis for T24* C1GALT1* KO models (Figure 6D; Figure S11; Table S6). A less intense band above 37 kDa (close to CD44s estimated molecular weight) was also obvious in glycoengineered models (Figure 6C), suggesting that *O*-glycans shortening may also impact on glycosites occupancy.

**Figure 7:**
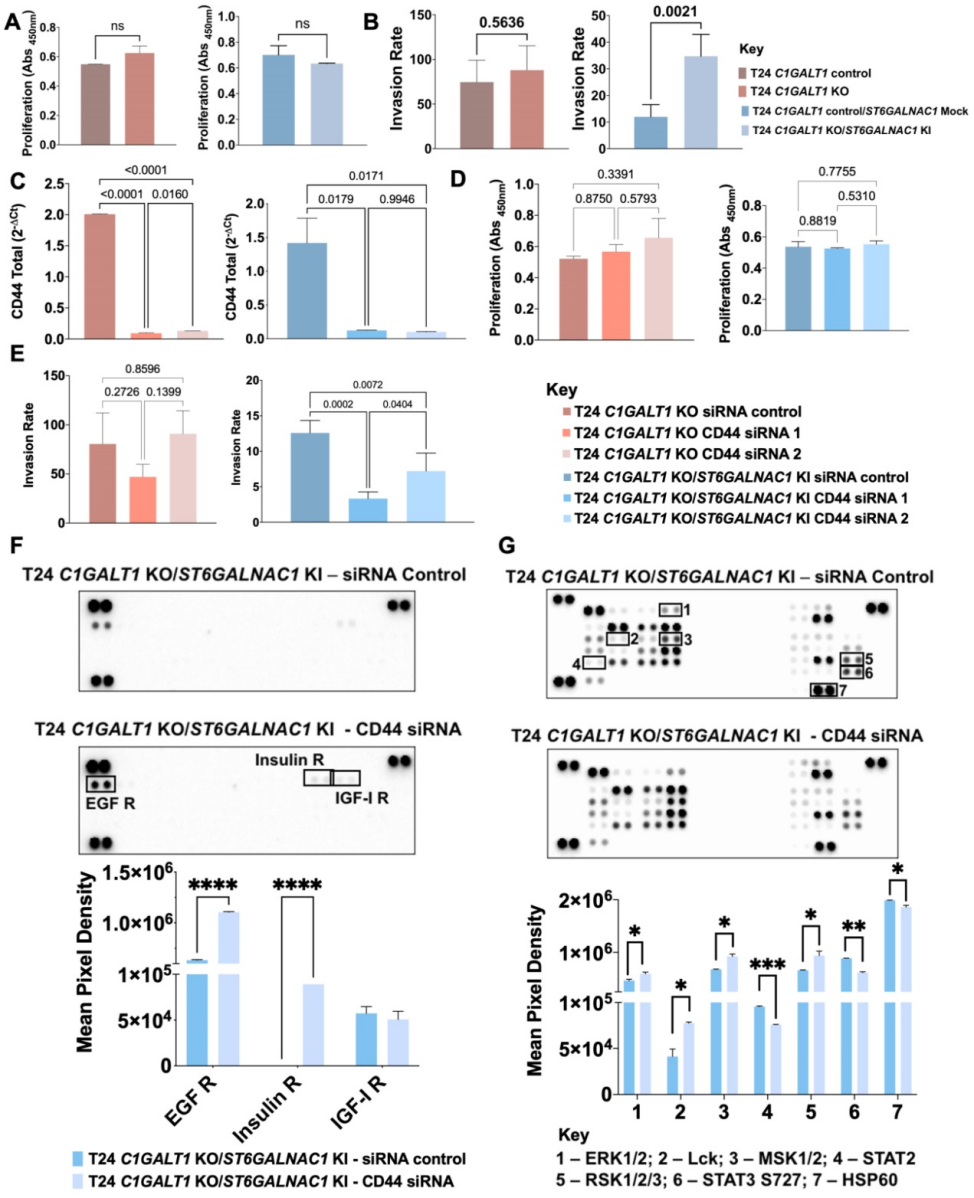
** CD44 glycosylation in CD44s-enriched cells shapes its functional contribution to proliferation and invasion. A)** *O*-glycans shortening by *C1GALT1* KO and *C1GALT1* KO/*ST6GALNAC*1 KI does not change the proliferation of T24 cells. T24 cells overexpressing Tn or STn antigens maintain proliferation rates compared to controls. **B)** Increased STn antigen at the cell surface, driven by *C1GALT1* KO and *ST6GALNAC*1 KI, enhances the invasive capacity of T24 cells. T24 cells overexpressing the Tn antigen do not change their capacity to invade Matrigel in comparison to the control *in vitro*. On the other hand, STn overexpression leads to a significant increase in invasion. **C)** CD44 silencing promotes a notable decrease in *CD44* gene expression in glycoengineered T24 cells expressing Tn and STn. *CD44* mRNA expression after siRNA is significantly decreased by approximately 95% and 91% in *C1GALT1* KO and *C1GALT1* KO*/ST6GALNAC1* KI T24 cells, respectively, in comparison to controls. **D)** CD44-Tn and STn glycoproteoforms do not play a role in T24 cells proliferation. After CD44s inhibition, *C1GALT1* KO and *C1GALT1* KO*/ST6GALNAC1* KI T24 cells maintained their proliferation in relation to controls, showing that CD44-Tn/STn glycoproteoforms do not influence cell proliferation. **E)** CD44-STn but not Tn glycoproteoforms drive invasion. After CD44s silencing, *C1GALT1* KO T24 cells maintained their capacity to invade Matrigel *in vitro*, whereas *C1GALT1* KO*/ST6GALNAC1* KI cells showed decreased invasion. Similar effects were observed in wild-type cells but not in Tn-expressing cells, suggesting that sialylation may have a role in tumor invasion.** F)** Influence of STn-overexpression on the response of tyrosine kinases receptors to CD44 silencing. We show an activation of RTK proteins such EGFR and Insulin receptor in the absence of CD44 for *C1GALT1* KO*/ST6GALNAC1* KI cells, opposite to the effect observed in wild-type cells. **G)** CD44-STn glycoproteoforms seem to promote the activation of relevant transcription factors involved in cell proliferation and invasion. In the absence of CD44, a significant downregulation of STAT3 S727 and STAT2 phosphorylation was observed in *C1GALT1* KO*/ST6GALNAC1* KI cells. Results correspond to the mean and standard deviation for three independent experiments. Triplicate measurements were conducted for each experiment. *P* values are presented for Unpaired T tests.

To disclose the influence of CD44 glycosylation, we induced a transient inhibition of the protein by siRNA in glycoengineered cell models and controls, which led to almost complete abrogation of *CD44* mRNA expression (Figure 7C), as previously observed for wild type cells. First, we confirmed that proliferation was not affected by alterations in glycosylation induced by *C1GALT1* KO and *ST6GALNAC1* KI (Figure 7A). Also, Tn overexpression did not change invasion, whereas STn-overexpression increased by 3-fold the number of T24 cells invading Matrigel (Figure 7B). The link between STn and invasion is in close agreement with our previous report for glycoengineered BC cells 29, and other cancer models showing increased cell motility and acquisition of invasive traits resulting from the presence of this antigen 47, 49, 81. Nevertheless, subsequent studies showed that T24 *C1GALT1* KO and *C1GALT1* KO*/ST6GALNAC1* KI cells proliferation was not significantly affected by CD44s silencing (Figure 7D). This contrasted with the anti-proliferative effect observed for T24 cells expressing more mature *O*-glycans under the same conditions (Figure 5C). Such findings suggest that proliferation may be fine-tuned by the nature of CD44s *O*-glycosylation, being activated by *O*-glycans extension. On the other hand, invasion induced by STn overexpression was reversed after CD44 inhibition (Figure 7E), as previously observed for wild type cells (Figure 5D). However, this effect was not so evident for Tn-expressing cells, suggesting that sialylation may play a role in CD44-mediated invasion, which warrants future confirmation. Collectively, these finds suggest that CD44-mediated invasion in T24 cell may be mostly driven by the nature of the splicing code.

We have then attempted to understand the functional impact of STn-overexpression on the activation of different RTKs upon siRNA-mediated knockdown of CD44. As shown in Figure 7F, CD44 silencing in STn positive cells induced a significant increase in activation of EGFR and Insulin receptors as well as of downstream proteins ERK 1/2, MSK 1/2 and RSK 1/2/3, all of which intermediates of the ERK/MAPK signaling pathway. Interestingly, wild type cells presented an opposite phenotype regarding activation of EGFR and insulin receptors under the same stimuli (Figure 5E). These findings again portrait the key role played by the glycocode in the regulation of oncogenic pathways governed by CD44. It further suggests that CD44-STn driven invasion may be supported by other molecular mechanisms. Additionally, we showed increased phosphorylation of Lck, a member of Src kinase family, which is intimately involved in T-cell activation in the absence of CD44 82, suggesting a broader role of CD44 in immune regulation that should be addressed in future studies.

Furthermore, we observed a significant downregulation of STAT3, STAT2 and HSP60 phosphorylation in STn-expressing cells upon CD44 inhibition (Figure 7G). STAT3 activation via CD44 has been linked to the acquisition of invasive traits and metastasis in different types of tumors 83, 84, suggesting a similar role in this context intimately linked to CD44. Altogether, these observations provide glances on the intricate nature of oncogenic pathways regulation by CD44, which should be carefully addressed by dedicated phosphoproteomics studies in the future. Moreover, it portrayed the key role played by glycosylation in CD44-mediated invasion of cancer cells. It suggests that these events may be modulated by sialylation of glycan chains, since this was not observed in Tn expressing cells. On the other hand, CD44 glycosylation appears to be key for defining the nature of oncogenic pathways supporting cancer aggressiveness.

In summary, we observed that invasion is mediated by the nature of CD44 splicing code and that cancer cells and tumors enriched for CD44s show higher invasion and worst prognosis. Furthermore, the splicing code presented by T24 cells was implicated in an onset of relevant oncogenic pathways that support these functional traits. Finally, we found that the nature of the CD44 glycocode is also crucial to enhance invasion and that CD44s-sTn/Tn glycoproteoforms hold tremendous potential to precisely target more aggressive subpopulation of cancer cells.

## Conclusions

CD44 is a pivotal glycoprotein in cancer, intricately related to most of its hallmarks, and widely accepted as a stem cell biomarker in BC 3, 16. The biological role of CD44 in cancer is intimately linked to the nature of protein isoforms 85 that, in the absence of dedicated analytical workflows, have been inferred from transcripts analysis complemented with immunoassays, lacking the necessary specificity for isoform distinction. Together with conflicting nomenclature, this has constituted a barrier for definitive CD44 exploitation in clinical settings. In fact, CD44 mRNA alternative splicing, which generates highly homologous polypeptide sequences and dense and heterogeneous glycosylation, has constituted a major obstacle for precise identification of cancer-specific glycoproteoforms and the establishment of solid structure-function relationships 2. Using BC as a model, we have started by exploring transcriptomics to provide the first glance at CD44 mosaicism in BC. This showed significant heterogeneity in terms of expressed isoforms and identified CD44s as a potential biomarker of invasion and poor prognosis. In agreement with these observations, CD44s was also strongly correlated with a subgroup of tumors displaying gene signatures characteristic of the basal BC molecular subtype but enriched for typical epithelial-to-mesenchymal transition markers. Later, we demonstrated that cells significantly enriched for CD44s displayed CD44-mediated invasion* in vitro*, reinforcing the close link between this isoform and disease progression and its potential for targeting more aggressive subpopulations of cancer cells. The focus should now be on providing unequivocal demonstrations about the functional role of CD44s in these cells. Interestingly, basal bladder tumors express high levels of genes typical of more undifferentiated urothelial basal cells (*KRT5*, *KRT6*, and* KRT14)* together with several transcription factors that support stem cell homeostasis and cancer progression also presented a CD44s^high^ phenotype. The association of EMT markers with CSC properties has been reported for more aggressive tumors 86, which may likely be also the case for BC. In support of this hypothesis, in a recent study, Zhu *et al.* has elegantly demonstrated the pivotal role played by CD44s as regulator of stem-like properties, invasion, and lung metastasis in BC, providing solid molecular grounds to our current observations in patient samples 87 Similar reports have been found for breast 88, lung 89, hepatic 90, and pancreatic 11 cancers, which supports the pan-carcinogenic nature of CD44s and its role in regulating EMT and adaptive plasticity of CSC. Nevertheless, transcriptomics also showed that CD44s expression may not be exclusive of tumors, as many healthy human organs may also express high levels of this isoform. This apparent lack of cancer specificity raises a major obstacle for precise cancer targeting, casting doubts on therapeutic development strategies. However, we showed that targeting CD44s *O*-glycoforms, such as the Tn and STn antigens associated with aggressive forms of cancer, may allow to overcome this limitation, since these glycoforms are rarely found in healthy tissues.

Additionally, we tackled the limitations of transcriptomics and proteomics for definitive CD44 splicing code characterization, bringing together these different omics and glycomics. We have demonstrated that CD44 characterization cannot be achieved by a single omics and highlighted the importance of adding glycosylation for definitive isoforms identification, materializing the concept of glycoproteogenomics 28. Overall glycoproteogenomics analysis of different cell models and bladder tumors supported transcriptomics data and provided a mass spectrometry-based approach for validation of several CD44 exon-exon junctions, moving one step forward in the characterization of this glycoprotein. It also confirmed CD44s as a major carrier of altered glycosylation linked to cancer aggressiveness, since we have demonstrated by mass spectrometry the presence of CD44s-(S)Tn in CD44s^high^/STn^high^ tumors. A major limitation of our glycoproteogenomics strategy was the incapacity to identify (glyco)peptides spanning multiple exon-exon junctions, which would be key for more definitive isoform characterization. Future studies should complement this approach with middle-down proteomics adopting partial protein digestions to generate larger and potentially more informative peptide fragments.

Then, exploring the functional implications of CD44 in cancer cells, we demonstrated that this glycoprotein may play a pivotal role driving cell proliferation and invasion. We also hypothesize that shorter splicing codes characterized by high CD44s levels may be a key factor driving invasion, which still warrants confirmation. Phosphoarray-based assays further reinforced the link between CD44 and key phospho-tyrosine kinases and related transcription factors, mainly associated to EGFR dependent signaling, supporting proliferation and invasion and providing relevant molecular grounds to understand its role in BC progression. Similar observations have been made for other cancer models including gastric 91, breast 92, head and neck squamous cell carcinoma 65 and lung 66 tumors, reinforcing the influence of CD44 as a co-regulator of RTK signaling and EGFR mediated pathways, thereby contributing to cell proliferation and invasion. Our study does not unequivocally demonstrate that CD44s is directly implicated in these processes, however it shows that it may be explored to target cells presenting these molecular features. Moreover, it this isoform maybe useful for identifying patients benefiting from therapies targeting these relevant oncogenic pathways, paving the way towards precision oncology.

Finally, exploring a library of glycoengineered cell models we have demonstrated that the nature of CD44 glycosylation in cells significantly enriched for CD44s is key to fine-tune its functional behavior, namely in terms of proliferation and invasion. CD44 is a heavily *O*-glycosylated protein, and it is likely that dramatic changes in glycans structures may induce major conformational changes implicating interactions with extracellular matrix components and cell surface receptors, which are yet to be fully understood 93. We found that CD44 inhibition in cells expressing high levels of sialylated *O*-glycans, including the STn antigen, had a major impact on cell invasion. Sialylation has long been described as a driver of cancer cells invasion, namely through the promotion of less cohesive cell phenotypes due to repulsive effects between proteins at the cell surface 94, 95. In fact, some reports suggest that sialylation could decrease the interaction of CD44 with its ligand hyaluronic acid, whose interaction contributes to cancer invasiveness and metastasis 25. By contrast, it has been previously demonstrated that CD44 *O*-glycans shortening increased hyaluronan binding capacity and may promote migratory pro-invasive cancer cell phenotypes in gastric cancer 93. Taken together, we propose that CD44 has a crucial role in cell invasion, however, it might be independent of the presence of STn moieties at its surface. On the other hand, a broader screening of main oncogenic pathways reflects another level of regulation dictated by the type of CD44 glycosylation, which appears to be key for defining oncogenic pathways adopted by cancer cells to support cancer aggressiveness. These findings, illustrate the relevance of CD44 glycocode characterization towards true precision oncology and casts important research topics for addressing CD44 role in cancer. It also supports the implementation of systems biology approaches, namely through an interrogation of the phosphoproteome for more comprehensive understanding about the role of this glycoprotein in cancer.

In summary, we have provided molecular and functional contexts linking CD44s and immature *O*-glycosylation, to cancer aggressiveness, in agreement with our previous reports linking both the protein and STn with invasion, metastasis and poor prognosis in BC 14, 34, 46. Therefore, we hypothesize that targeting CD44-Tn/STn glycoproteoforms may constitute a key strategy to control BC progression, which will be addressed in future studies. Given that CD44s and STn are frequently found in many human tumors linked to unfavorable prognosis 46, 96, we also believe that this may constitute a valuable cancer signature for future clinical interventions at different levels. Moreover, according to the analysis of healthy tissues, CD44s glycosignatures are highly cancer specific, holding tremendous potential to overcome some of the off-target limitations associated with glycans. Therefore, this study may come to contribute significantly to the development of antibodies and other ligands against cancer cells, enabling the creation of an onset of novel theranostics interventions (cancer detection, targeted therapeutics) and decisively aid patient stratification. For instance, encouraging pre-clinical studies were presented concerning CAR-Ts targeting Tn and STn antigens in MUC1 97, which may now be adapted for more cancer specific proteoforms such as CD44-Tn/STn. Moreover, it may pave the way for glycan-based cancer vaccines capable of educating the immune system to respond to abnormally glycosylated cancer cells, avoiding cancer spread and relapse through protective memory. Overall, we have provided a roadmap to characterize the functional role of glycosylation in CD44, paving the way for more educated clinical interventions. This analytical strategy sets the grounds for a comprehensive characterization of many different types of tumors, constituting a crucial step towards understanding of the role of CD44 and other clinically relevant cell surface proteins in health and disease.

## Supplementary Material

Supplementary figures and tables.

## Figures and Tables

**Table 1:** Peptides corresponding to CD44 exon and exon-exon junctions identified by SDS-PAGE-nanoLC-MS/MS.

Mw (kDa)	5637		T24	
	Exons and exon-exon junctions^1^	Assignment^2^ ^(Protein coverage)^	Exon and exon-exon junctions^1^	Assignment^2^ ^(Protein coverage)^
>200-250	e1-e2; e2-e3; e3-e4; e4-e5; v2-v3, v3-v4; v4-v5; v6 (partial); v10-e15; e19; CD44sol^3^	CD44v2-10 (46%) CD44 sol (96%)	No ID	-
200-150	No ID	-	No ID	-
150-100	e1-e2; e2-e3; e3-e4; e4-e5; v2-v3, v3-v4; v4-v5; v6 (partial); v10-e15; e19; e5-e15	CD44v2-10 (49%) CD44s (59%)	CD44sol^3^	CD44sol (96%)
100-75	e1-e2; e2-e3; e3-e4; e4-e5; v2-v3; v3-v4; v6; v10-e15; e5-e15; e19, CD44sol^3^	CD44v2-10 (43%) CD44s (68%) CD44sol (95%)	e1-e2; e2-e3; e3-e4; e4-e5; v2-v3, v3-v4; v4-v5; v7 (partial); v10-e15; e19; e5-e15, CD44sol^3^	CD44v2-10 (48%) CD44s (58%) CD44sol (96%)
75-50	No ID	-	e1-e2; e2-e3; e3-e4; e5-v3; v2-v3, v3-v4, v4-v5; v10-e15, e19, e5-e15; CD44sol^3^	CD44v2-10 (47%) CD44v3-10 (49%) CD44s (73%) CD44sol (96%)
50-37	No ID	-	e1-e2, e2-e3, e3-e4, e4-e5, e5-e15	CD44s (59%)
37-25	e1-e2; e2-e3; e3-e4; e4-e5; v2-v3; e5-v3; v3-v4; v10-e15, e19; e5-e15, CD44sol	CD44v2-10 (49%) CD44v3-10 (50%) CD44s (59%) CD44sol (97%)	e1-e2; e2-e3; e3-e4; e4-e5; v2-v3, v3-v4; v4-v5; v6 (partial); v7 (partial); v10-e15; e19; e5-e15	CD44v2-10 (49%) CD44s (58%)
25-20	No ID	-	No ID	-
20-15	No ID	-	No ID	-
15-10	No ID	-	No ID	-

^1^Given the high coverage and significantly different structure, we use the general designation to highlight CD44sol; ^2^Protein coverages considers the complete variant deduced from e5-variable region junctions, without considering exon-exon jumps; ^3^CD44sol was used as a general designation for the following exon-exon junctions: v2(partial)-v3(partial), v3(partial)-v4(partial), v4(partial)-v5(partial), v5(partial)-v6(partial), v6(partial)-v10(partial), v10(partial)-e16(partial), whereas partial stands for incomplete exon sequences.

## Abbreviations

B2M: β-2-microglobulin
BC: bladder cancer
BLCA: Bladder Carcinoma
BSA: bovine serum albumin
CD44: cluster of differentiation 44
CD44s: CD44 standard splicing isoform
CID: collision induced fragmentation
CSC: cancer stem cells
DAPI: 4',6'-diamidino-2-phenylindole dihydrochloride
DTT: 1,4-dithiothreitol
EGFR: epidermal growth factor receptor
EMT: epithelial-to-mesenchymal transition
FFPE: formalin-fixed paraffin-embedded
GTEx: genotype-tissue expression
HPRT: Hypoxanthine-guanine phosphoribosyltransferase
IGF-1R: insulin-like growth factor 1 receptor
IP: immunoprecipitation
IR: insulin receptor
KI: knock-in
KO: knock-out
MALT: mucosa-associated lymphoid tissue
MIBC: muscle invasive bladder cancer
nanoLC-MS/MS: nano liquid chromatography mass spectrometry
NCE: normalized collision energy
NMIBC: non-muscle invasive bladder cancer
PFA: paraformaldehyde
PLA: proximity ligation assay
RT-PCR: real time-polymerase chain reaction
RT: room temperature
RTK: receptor tyrosine kinase
SiRNA: small interfering RNA
STn: sialyl-Tn
VVA: *vicia villosa* lectin
WB: western blotting
