# Supplementary material for: Glycoproteogenomics characterizes the CD44 splicing code associated with bladder cancer invasion
Source: Theranostics. 2022 Mar 28;12(7):3150–77. doi: 10.7150/thno.67409 (PMC9065180; doi:10.7150/thno.67409)
Supplement: Supplementary file 1 — Supplementary figures and tables. [file thnov12p3150s1.pdf]

Glycoproteogenomics characterizes the CD44 splicing code associate with bladder cancer invasion

-Supplementary figures and tables-

Cristiana Gaiteiro[1,2,3,4,5], Janine Soares[1,2,3,4,6], Marta Relvas-Santos[1,2,3,4,7,8,9], Andreia Peixoto[1,2,3,7,8], Dylan Ferreira[1,2,3,4,5,7,8,], Paula Paulo[2,3,10], Andreia Brandão[2,3,10], Elisabete Fernandes[1,2,3,11], Rita Azevedo[12], Carlos Palmeira[1,2,3,11,13], Rui Freitas[1,2,3,4,7,8], Andreia Miranda[1,2,3,14], Hugo Osório[7,14,15], Jesús Prieto[5], Luís Lima[1,2,3], André M. N. Silva[9,16], Lúcio Lara Santos[1,2,3,4,11,16,17], José Alexandre Ferreira[1,2,3,4,16,a]

[1]Experimental Pathology and Therapeutics Group, IPO Porto Research Center (CI-IPOP), Portuguese Oncology Institute (IPO Porto), 4200-072 Porto, Portugal; [2]RISE@CI-IPOP (Health Research Network), Portuguese Oncology Institute of Porto (IPO Porto), 4200-072 Porto, Portugal; [3]Porto Comprehensive Cancer Center (P.ccc), 4200-072 Porto, Portugal; [4]Institute of Biomedical Sciences Abel Salazar (ICBAS), University of Porto, 4050-013 Porto, Portugal; [5]Center for Applied Medical Research (Centro de Investigación Médica Aplicada, CIMA), University of Navarra, 31008 Pamplona, Navarra, Spain; [6]REQUIMTE-LAQV, Department of Chemistry, University of Aveiro, 3810-193 Aveiro, Portugal; [7]Institute for Research and Innovation in Health (i3S), University of Porto, 4200-135 Porto, Portugal; [8]Institute for Biomedical Engineering (INEB), University of Porto, 4200-135 Porto, Portugal; [9]REQUIMTE-LAQV, Department of Chemistry and Biochemistry, Faculty of Sciences of the University of Porto, 4169-007 Porto, Portugal; [10]Cancer Genetics Group, IPO Porto Research Center (CI-IPOP), Portuguese Oncology Institute (IPO Porto), 4200-072 Porto, Portugal; [11]FP-I3ID, University Fernando Pessoa, 4249-004 Porto, Portugal; [12]Laboratoire d'Etude du Métabolisme des Médicaments (LEMM), CEA, INRA, Université Paris Saclay, F-91191, Gif-sur-Yvette cedex, France; [13]Immunology Department, Portuguese Oncology Institute (IPO Porto), 4200-072 Porto, Portugal; [14]Faculty of Medicine of the University of Porto, 4200-319 Porto, Portugal; [15]Ipatimup—Institute of Molecular Pathology and Immunology of the University of Porto, University of Porto, 4200-135 Porto, Portugal; [16]GlycoMatters Biotech, 4500-162 Espinho, Portugal; [17]Department of Surgical Oncology, Portuguese Oncology Institute (IPO Porto), 4200-072 Porto, Portugal

[a]Corresponding author:

José Alexandre Ferreira (jose.a.ferreira@ipoporto.min-saude.pt)

ORCID ID: https://orcid.org/0000-0002-0097-6148

Experimental Pathology and Therapeutics Group, Research Centre, Portuguese Oncology Institute of Porto, R. Dr. António Bernardino de Almeida 62, 4200-072 Porto, Portugal; Tel. +351 225084000 (ext. 5111).

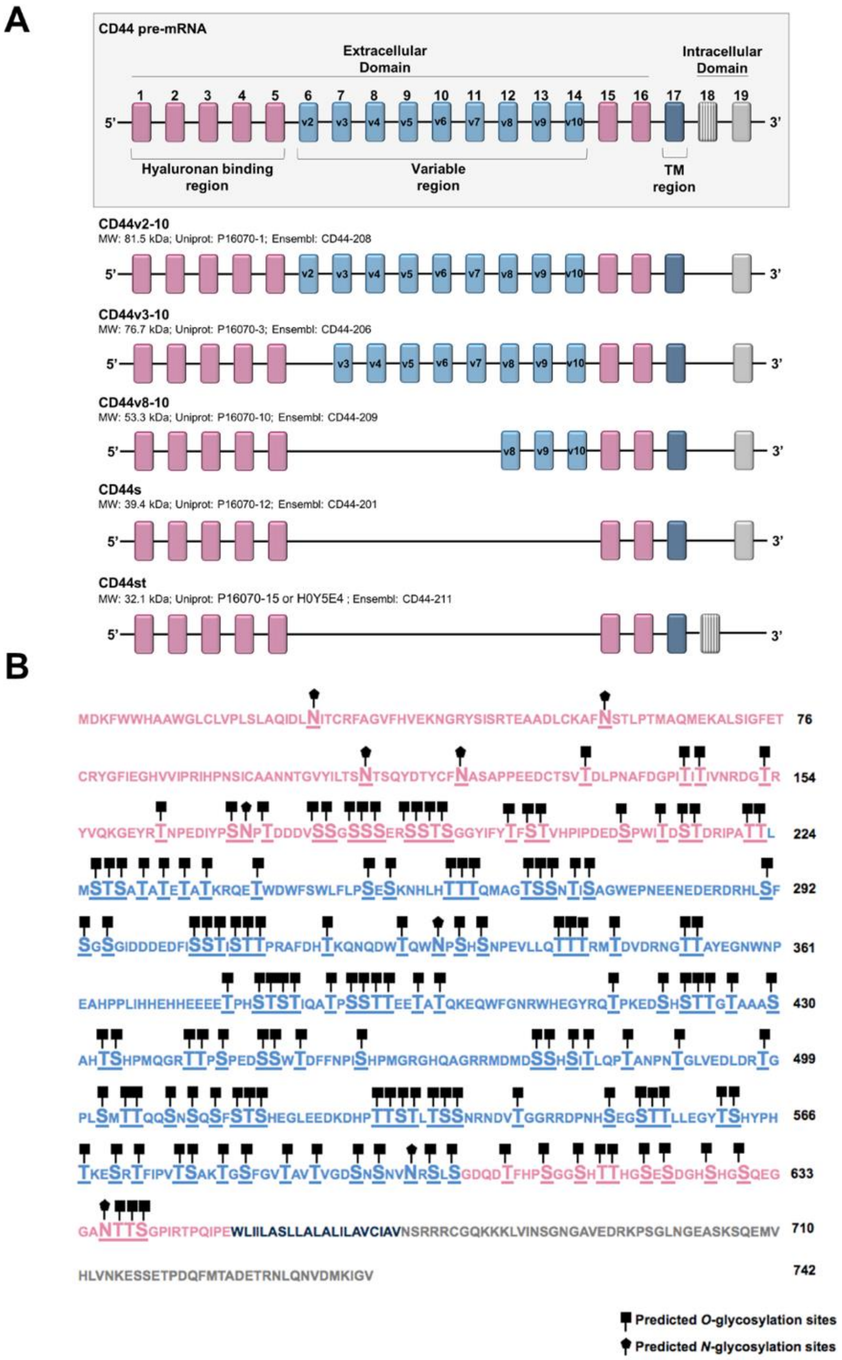

Figure S1. A) Schematic representation of human CD44 pre-mRNA and experimentally confirmed isoforms resulting from alternative splicing and extensively explored in this study. Exons generally regarded as conserved are represented in pink (constitutive exons e1-e5 and e15-e16), variable exons are represented in blue (exons v6-v14), the transmembrane region is represented in dark blue (exon e17), and the intracellular tail is represented in grey (exons e18-e19). Exon 18, filled with gray stripes, contains an early 3'UTR and is only present in the CD44st isoform. The estimated molecular weight for each variant (without posttranslational modifications), Uniprot and Ensemble codifications are also highlighted. **B) Amino acid sequence evidencing potential *O*- and *N*-glycosites.** *O*-glycosylation sites were predicted using the Net*O*Glyc 4.0 server (http://www.cbs.dtu.dk/services/NetOGlyc/) and *N*-glycosylation sites with the Net*N*Glyc 1.0 server (http://www.cbs.dtu.dk/services/NetNGlyc/). The different domains

of the protein are highlighted using the same color code as in panel A and evidences the high density of *O*-glycosites in comparison to *N*-glycosites, which is particularly evident in the variable region.

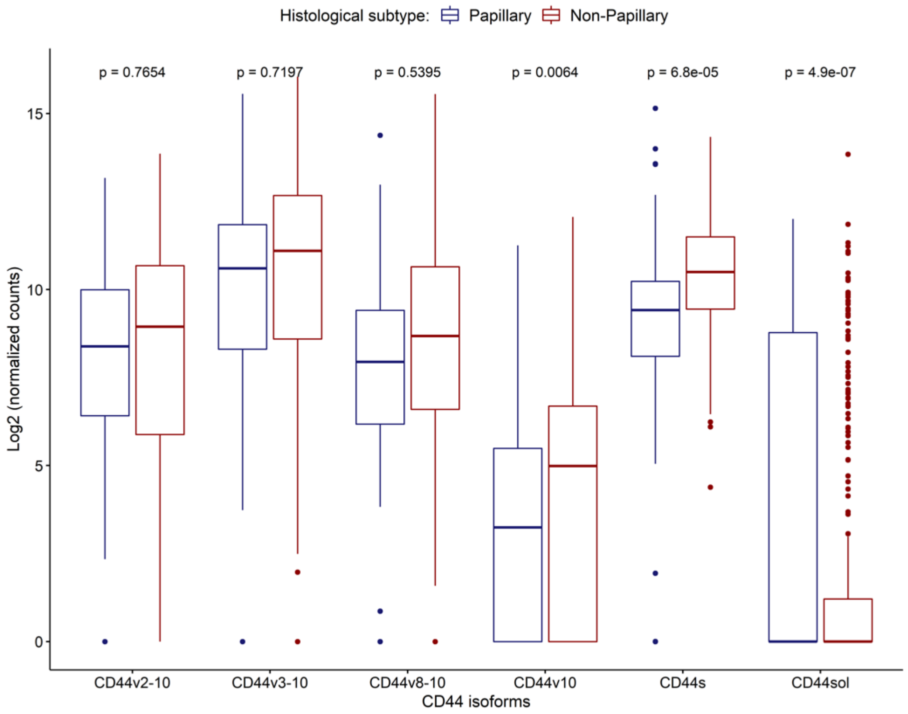

Figure S2. CD44 variants expression according to the histological subtypes of TCGA muscle invasive bladder tumors. Wilcoxon test revealed a significant overexpression of CD44s in more aggressive non-papillary lesions.

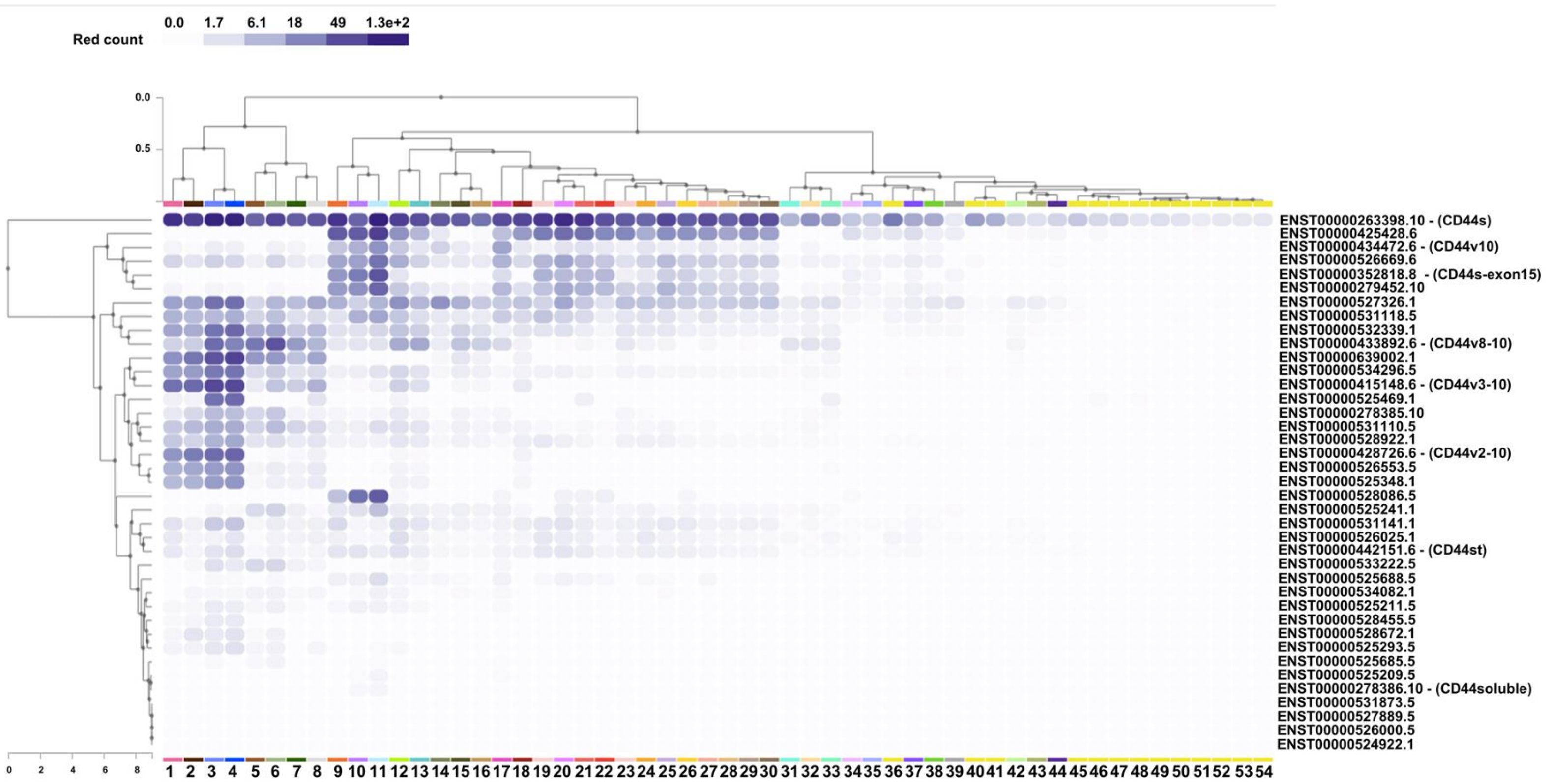

KEY

1- Vagina; 2- Esophagus-Mucosa; 3- Skin – Sun Exposed (Lower leg); 4- Skin – Not Sun Exposed (Suprapubic); 5- Pancreas; 6- Minor Salivary Gland; 7- Thyroid; 8- Prostate; 9- Adipose – subcutaneous; 10- Cells- EBV-transformed lymphocytes; 11- Cells- Cultured fibroblasts; 12- Lung: 13- Breast – Mammary Tissue; 14- Spleen; 15- Small Intestine – Terminal Ileum; 16- Colon – Transverse; 17- Whole Blood; 18- Bladder; 19- Cervix – Ectocervix; 20- Uterus; 21- Artery – Aorta; 22- Artery – Tibial; 23- Fallopian Tube; 24- Adipose – Visceral (Omentum); 25- Cervix – Endocervix; 26- Nerve – Tibial; 27- Artery – Coronary;28- Colon – Sigmoid; 29- Esophagus – Muscularis; 30- Esophagus – Gastroesophageal Junction; 31- Kidney – Cortex; 32- Stomach; 33- Kidney – Medulla; 34- Ovary; 35- Muscle – Skeletal; 36- Brain – Spinal cord (cervical c-1); 37- Heart – Atrial Appendage; 38- Adrenal Gland; 39- Testis; 40- Brain – Substantia nigra; 41- Brain – Hypothalamus; 42- Pituitary; 43- Liver; 44- Heart – Left Ventricle; 45- Brain – Hippocampus; 46- Brain – Cerebellum; 47- Brain – Amygdala; 48- Brain – Cortex; 49- Brain – Cerebellar Hemisphere; 50- Brain – Caudate (basal ganglia); 51- Brain – Frontal Cortex (BA9); 52- Brain – Nucleus accumbens (basal ganglia); 53- Brain – Putamen (basal ganglia); 54- Brain – Anterior cingulate cortex (BA24).

Figure S3. CD44 splice variants expression in healthy human tissues. Transcriptomics analysis of healthy tissues for CD44 isoforms were obtained and adapted from the GTEx Portal on 03/26/21 and/or dbGaP accession number phs000424.v8.p2. The Figure shows the complex mosaicism of CD44 mRNA in healthy tissues, including the co-expression of multiple isoforms in the same tissue. It highlights that CD44s is not expressed in healthy organs. Moreover, CD44st that differs from CD44s in terms of cytoplasmic tail but presents a similar extracellular domain was detected in low abundance in a restricted number of cells/organs, mostly from the skin, fibroblasts, subcutaneous adipocytes, the female reproductive system, the esophagus and breast. On the other hand, CD44s related structures lacking exon 15 (CD44s-exon 15) was present in high abundance in adipose tissues, fibroblasts, lungs, blood cells, across the female reproductive system and the gastrointestinal tract.

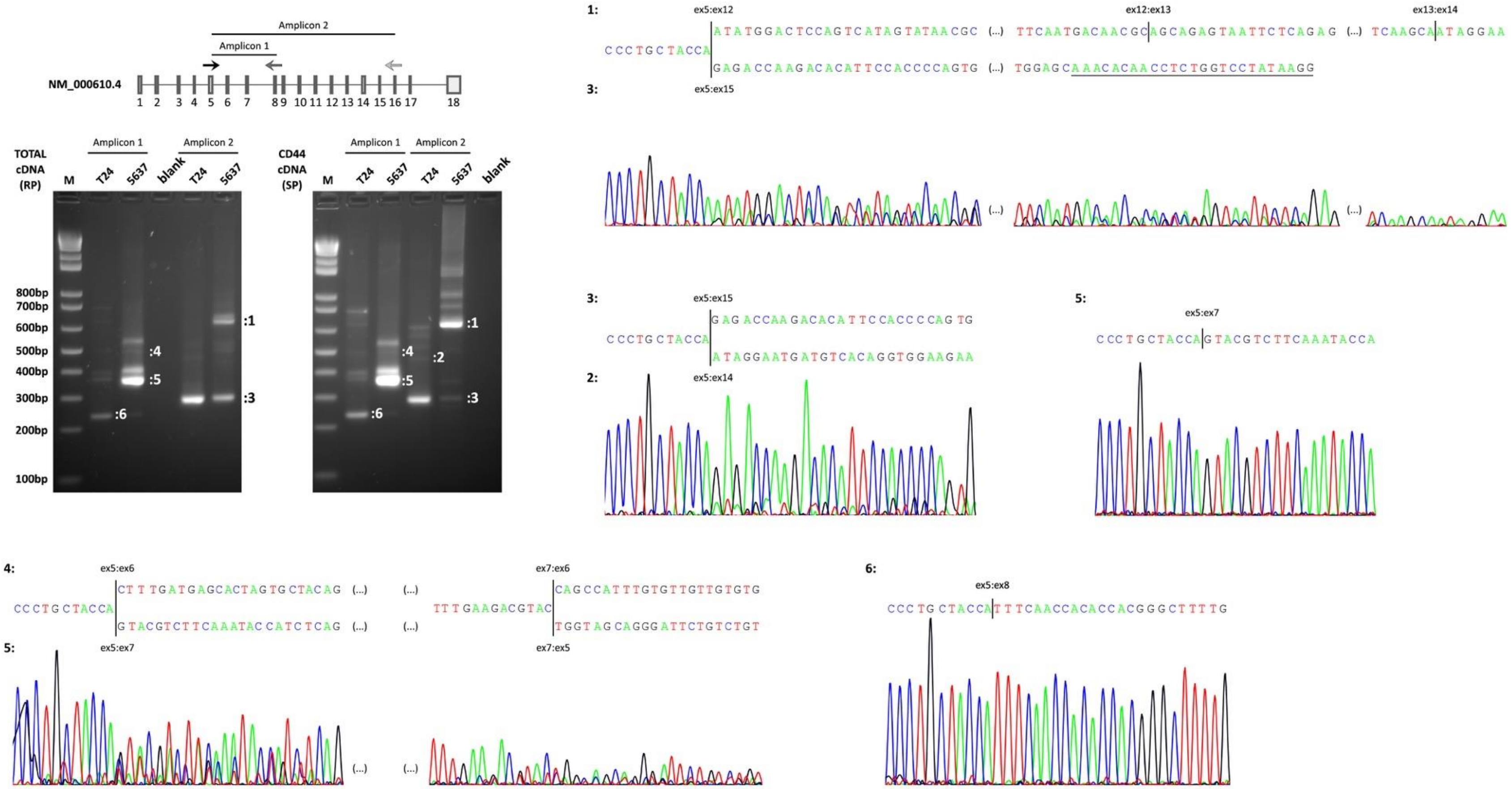

Figure S4. Reverse Transcriptase-Polymerase Chain Reaction and Sanger Sequencing for the *CD44* gene using random primer (RP) and e16 specific primer amplification. Two amplicons have been used: amplicon 1: e5-v4; amplicon 2: e5-e16. According to these experiments, 5637 were enriched longer isoforms in relation to T24 cells. However, we could not identify the precise nature of the long isoforms by sanger sequencing, most likely because of mixture of different isoforms. Supporting the distinct splicing codes presented by these cell lines, the following differences were observed: **e5-v8-v9-v10-(…)-e16** (**band 1**)**:** 5637 (main band)>T24; **e5-v10-(…)-e16** (**band 2**)**:** only in T24; **e5-e15-(…)-e16** (**band 3**)**:** T24 (main band; most likely corresponding to CD44s and/or st)>5637; **e5-v2-v3-(…)-e16** (**band 4**)**:** only in 5367; **e5-v3-v4-(…)-e16** (**band 5**)**:** 5637>T24; **e5-v4-(…)-e16** (**band 6**)**:** only in T24.

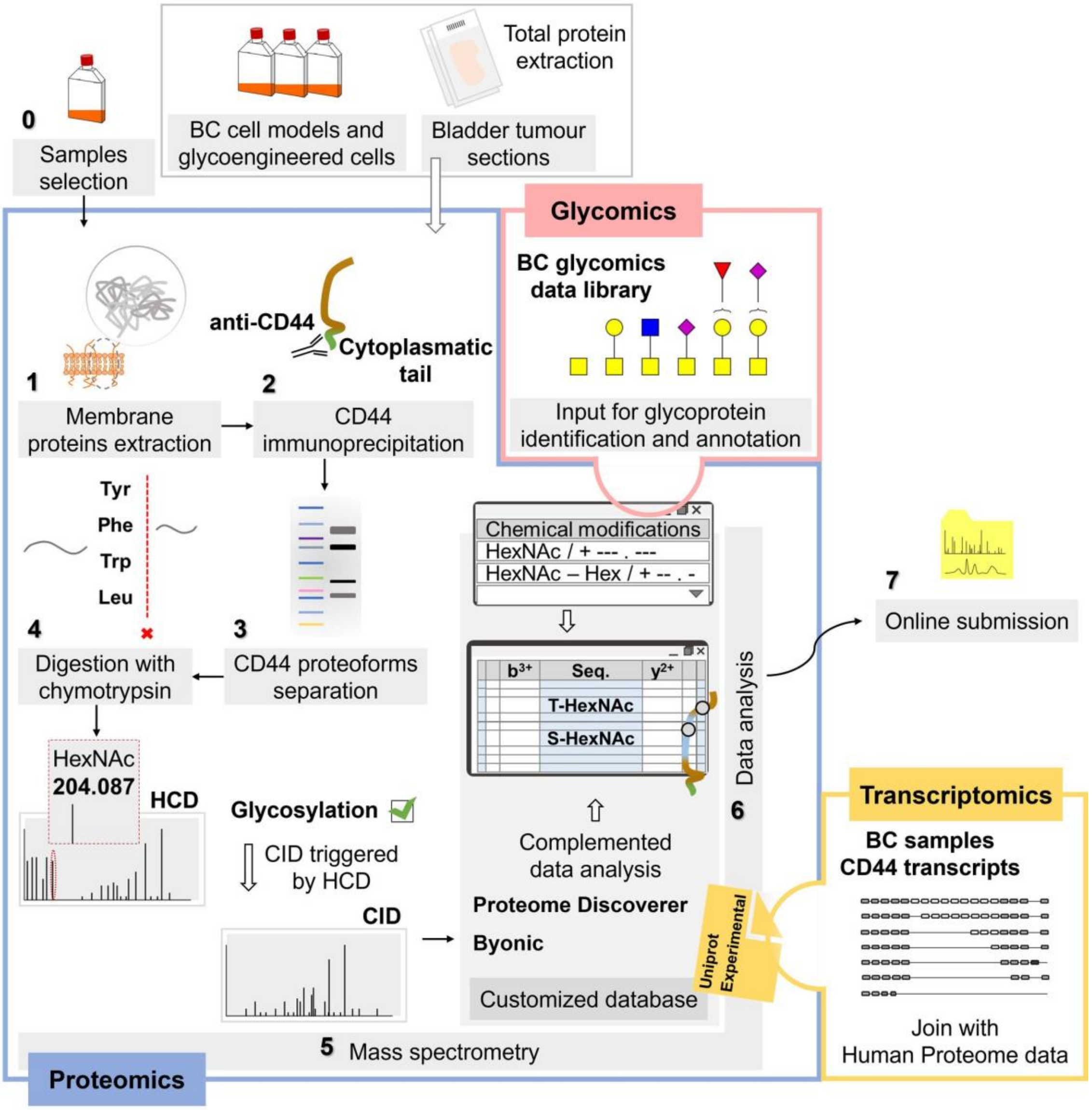

Figure S5. Glycoproteogenomics workflow for identification of CD44 glycoproteoforms. Briefly, bladder cancer cell models, glycoengineered cells and tumor tissue sections were used as starting material. **1.** Membrane glycoproteins from cell lines are isolated by differential ultracentrifugation. **2.** CD44 is immunoprecipitated using a monoclonal antibody targeting the cytoplasmatic tail domain expressed by most CD44 isoforms. **3.** CD44-IP enriched extracts were then digested with sialidase, separated by gradient SDS-PAGE electrophoresis. **4.** Bands are excised from the gels, proteins are reduced, alkylated, and digested with chymotrypsin *in* gel. **5.** Chymotrypsin digests are analyzed by nanoLC-MS/MS (HCD-MS2 and CID-MS2 with a dependent acquisition based on HCD detection of HexNAc oxonium ion at *m/z* 204.087. **6.** Glycoproteoforms identification using a glycoproteogenomics approach. RNAseq was used to identify CD44 isoforms expressed by 5637 and T24 cells. Predicted protein sequences were included in a database together with the human proteome from Uniprot database and used for protein identification in the Proteome Discoverer bioinformatics platform. Glycomics analysis was performed in parallel to determine the glycan structures that may be present as variable CD44 post-translational modification. This information was included upon protein identification and glycopeptides annotation by MS/MS.

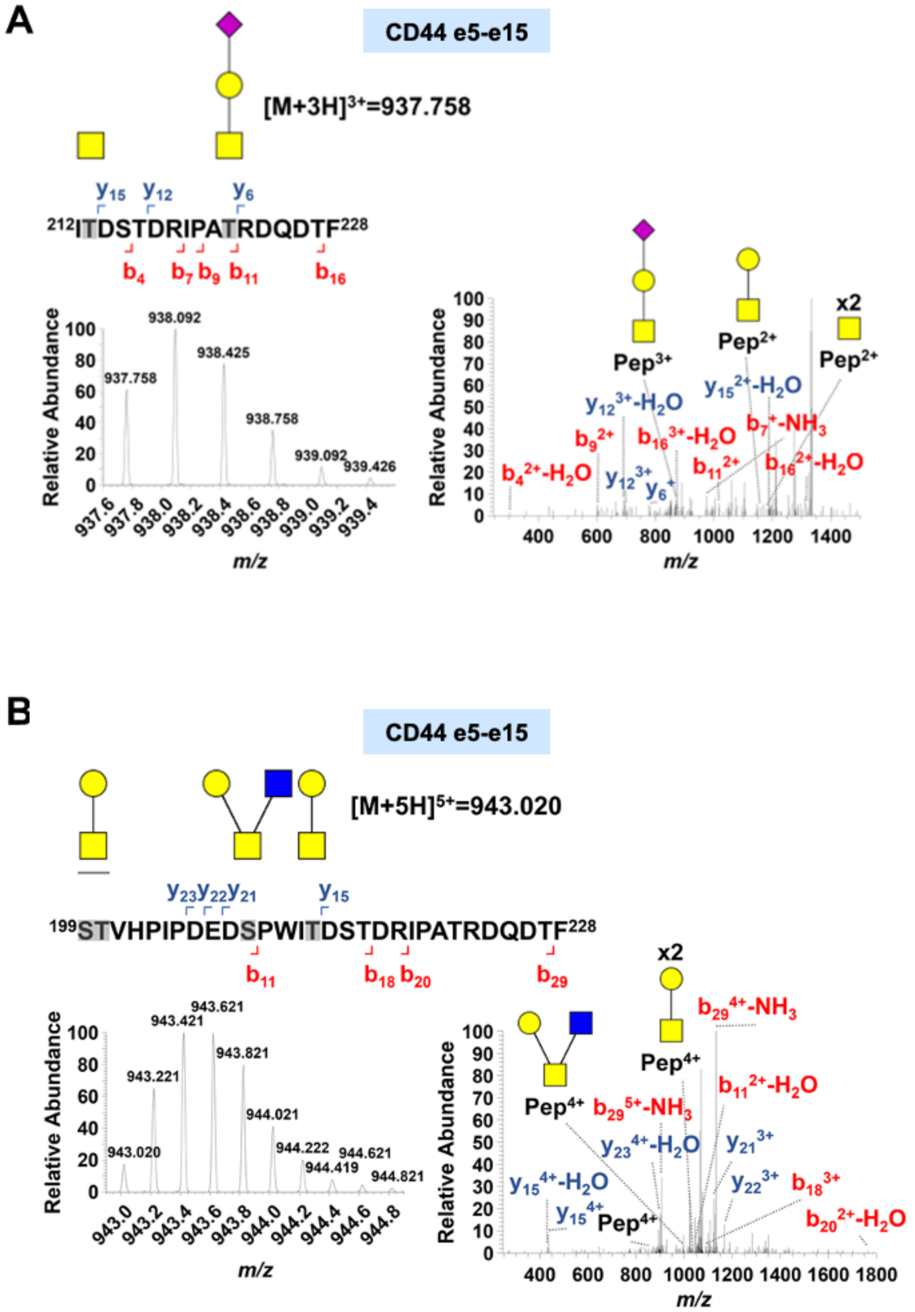

Figure S6. CID spectra for CD44s glycopeptides carrying *O*-glycans identified in T24 cells. A) CID spectrum for CD44s-Tn/ST and B) CD44s-T/core 2 glycopeptides. CD44s glycopeptides carrying these *O*-glycans were identified by CID based on an HCD data dependent acquisition. Briefly, species presenting HCD product ion spectrum with an HexNAc fragment at m/z 204.087 were elected for CID fragmentation. CID spectra confirmed the glycan structures and presented several *y*- and *b*-series peptide fragments that helped supporting glycosites assignment (highlighted in grey in the peptide sequence). Collectively, these spectra confirm the presence of the illustrated *O*-glycans in CD44s from T24 cells and the heterogeneous nature of glycosylation at the peptide level.

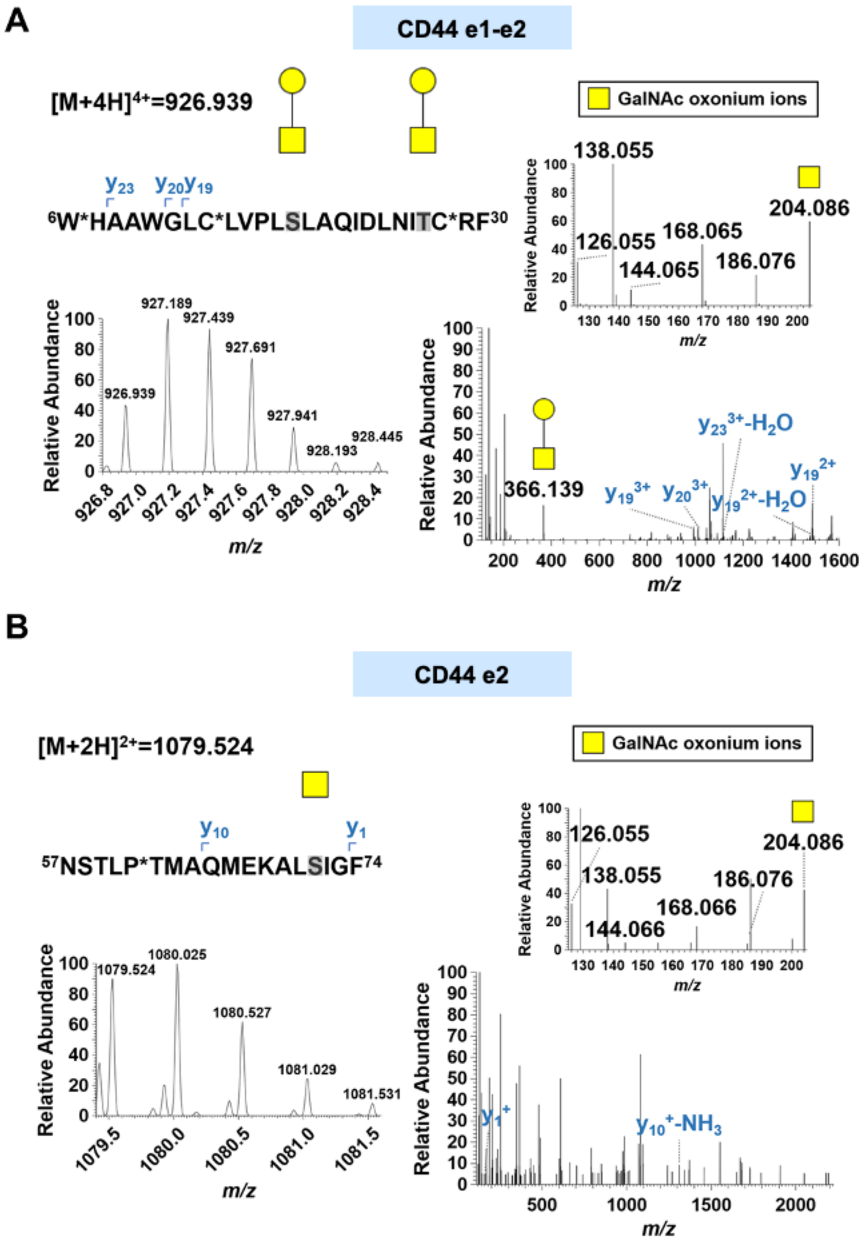

Figure S7. A) CD44-T and B) Tn glycopeptides belonging to the conserved extracellular domain in different isoforms identified in muscle invasive bladder tumors by nanoLC-HCD-MS/MS. Both MS/MS spectra present the HexNAc oxonium ions, namely the ion at *m/z* 204.086. The MS/MS spectrum of the CD44-T glycopeptide also presents an evident ion at *m/z* 366.139, corresponding to the oxonium ion for the T antigen. y-series ions resulting from peptide backbone fragmentation were also highlighted. The sequence of the glycopeptide presents the glycosites identified in grey. The symbol *corresponds to amino acid modifications: W – hydroxykynurenine; C – carbamidomethyl; P – glutamic semialdehyde.

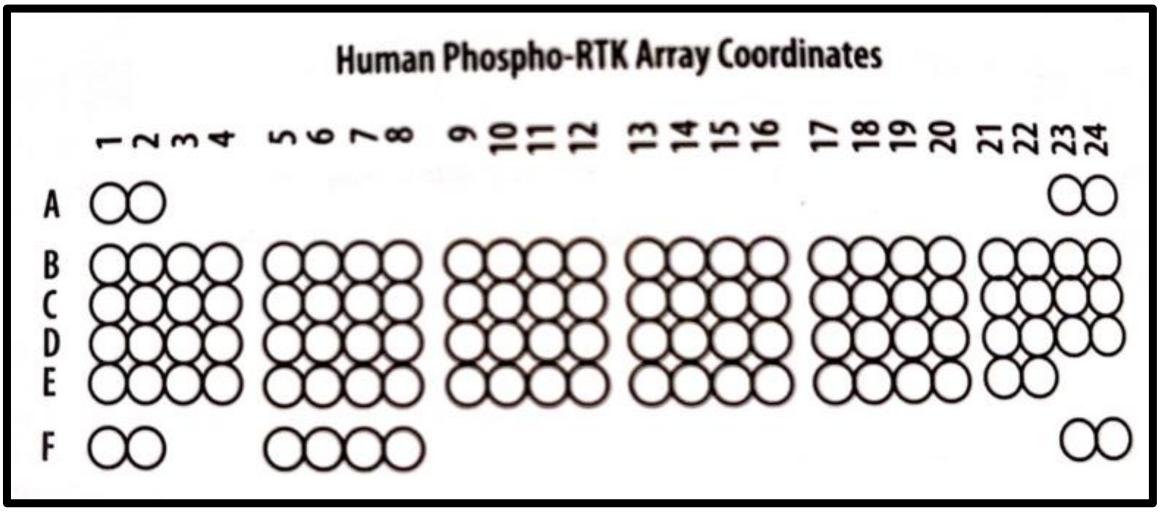

Coordinate	Receptor Family	RTK/Control
**A1, A2**	Reference Spots	-
**B1, B2**	Reference Spots	-
**B3, B4**	EGF R	EGF R
**B5, B6**	EGF R	ErbB2
**B7, B8**	EGF R	ErbB4
**B9, B10**	FGF R	FGF R1
**B11, B12**	FGF R	FGF R2α
**B13,B14**	FGF R	FGF R3
**B15, B16**	FGF R	FGF R4
**B17, B18**	Insulin R	Insulin R
**B19, B20**	Insulin R	IGF-I R
**B21, B22**	Axl	Axl
**B23, B24**	Axl	Dtk
**C1, C2**	Axl	Mer
**C3, C4**	HGF R	HGF R
**C5, C6**	HGF R	MSP R
**C7, C8**	PDGF R	PDGF Rα
**C9, C10**	PDGF R	PDGF Rβ
**C11, C12**	PDGF R	SCF R
**C13, C14**	PDGF R	Flt-3
**C15, C16**	PDGF R	M-CSF R
**C17, C18**	RET	c-Ret
**C19, C20**	ROR	ROR1
**C21, C22**	ROR	ROR2
**C23, C24**	Tie	Tie-1
**D1, D2**	Tie	Tie-2
**D3, D4**	NGF R	TrkA
**D5, D6**	NGF R	TrkB
**D7, D8**	NGF R	TrkC
**D9, D10**	VEGF R	VEGF R1
**D11, D12**	VEGF R	VEGF R2
**D13, D14**	VEGF R	VEGF R3
**D15, D16**	MuSK	MuSK

**D17, D18**	Eph R	EphA1
**D19, D20**	Eph R	EphA2
**D21, D22**	Eph R	EphA3
**D23, D24**	Eph R	EphA4
**E1, E2**	Eph R	EphA6
**E3, E4**	Eph R	EphA7
**E5, E6**	Eph R	EphB1
**E7, E8**	Eph R	EphB2
**E9, E10**	Eph R	EphB4
**E11, E12**	Eph R	EphB6
**E13, E14**	Insulin R	ALK
**E15, E16**	-	DDR1
**E17, E18**	-	DDR2
**E19, E20**	Eph R	EphA5
**E21, E22**	Eph R	EphA10
**F1, F2**	Reference spots	-
**F5, F6**	Eph R	Ephb3
**F7, F8**	-	RYK
**F23, F24**	Control (-)	PBS

Figure S8. The human phospho-receptor tyrosine kinase (Phospho-RTK) array coordinates. The phospho-array presents capture and control antibodies spotted in duplicates for the 49 different phosphorylated human RTKs, which are represented by alpha-numeric coordinates.

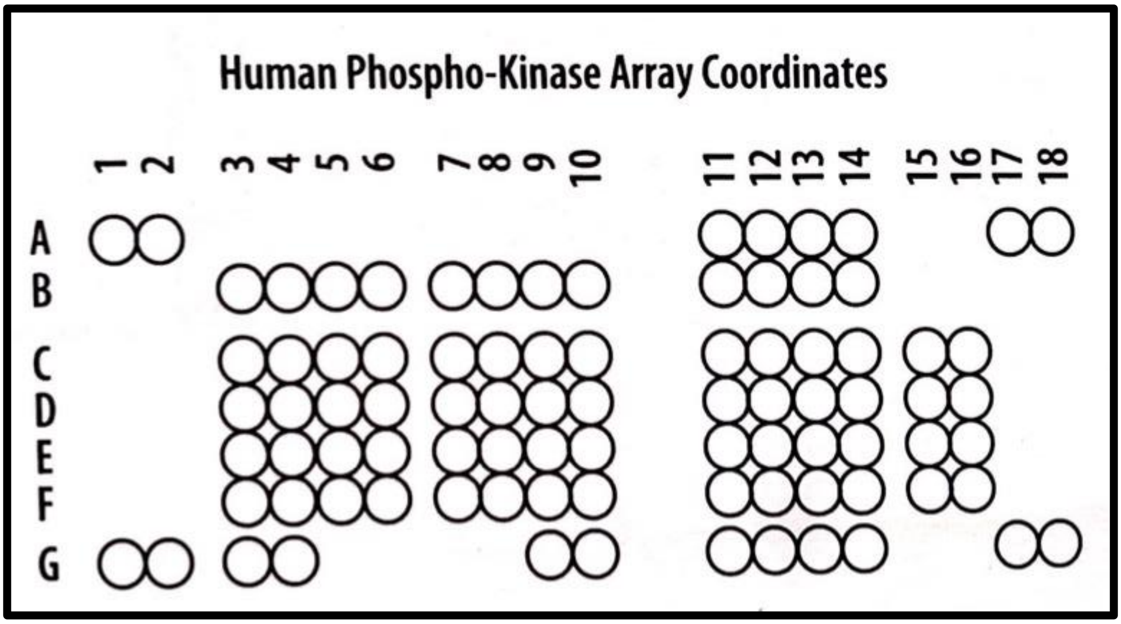

Coordinate	Target/Control	Phosphorylation Site
**A1, A2**	Reference Spot	-
**A11,A12**	Akt 1/2/3	T308
**A13, A14**	Akt 1/2/3	S473
**A17, A18**	Reference Spot	-
**B3, B4**	CREB	S133
**B5, B6**	EGF R	Y1086
**B7, B8**	eNOS	S1177
**B9, B10**	ERK 1/2	T202/Y204, T185/Y187
**B11, B12**	Chk-2	T68
**B13, B14**	c-Jun	S63
**C3, C4**	Fgr	Y412
**C5, C6**	GSK-3α/β	S21/S9
**C7, C8**	GSK-3β	S9
**C9, C10**	HSP27	S78/S82
**C11, C12**	p53	S15
**C13, C14**	p53	S46
**C15, C16**	p53	S392
**D3, D4**	JNK 1/2/3	T183/Y185, T221/Y223
**D5, D6**	Lck	Y394
**D7, D8**	Lyn	Y397
**D9, D10**	MSK 1/2	S376/S360
**D11, D12**	p70 S6 Kinase	T389
**D13, D14**	p70 S6 Kinase	T421/S424
**D15, D16**	PRAS40	T246
**E3, E4**	p38α	T180/Y182
**E5, E6**	PDGF Rβ	Y751
**E7, E8**	PLC-γ1	Y783
**E9, E10**	Src	Y419
**E11, E12**	PYK2	Y402
**E13, E14**	RSK 1/2	S221/S227

**E15, E16**	RSK 1/2/3	S380/S386/S377
**F3, F4**	STAT2	Y689
**F5, F6**	STAT5a/b	Y694/Y699
**F7, F8**	WNK1	T60
**F9, F10**	Yes	Y426
**F11, F12**	STAT1	Y701
**F13, F14**	STAT3	Y705
**F15, F16**	STAT3	S727
**G1, G2**	Reference Spot	-
**G3, G4**	β-Catenin	-
**G9, G10**	PBS (Control -)	-
**G11, G12**	STAT6	Y641
**G13, G14**	HSP60	-
**G17, G18**	PBS (Control -)	-

Figure S9. The human phospho-kinase array coordinates. The phospho-array presents capture and control antibodies spotted in duplicates for the detection of the relative levels of phosphorylation of 37 kinase phosphorylation sites and 2 related total proteins represented by alpha-numeric coordinates.

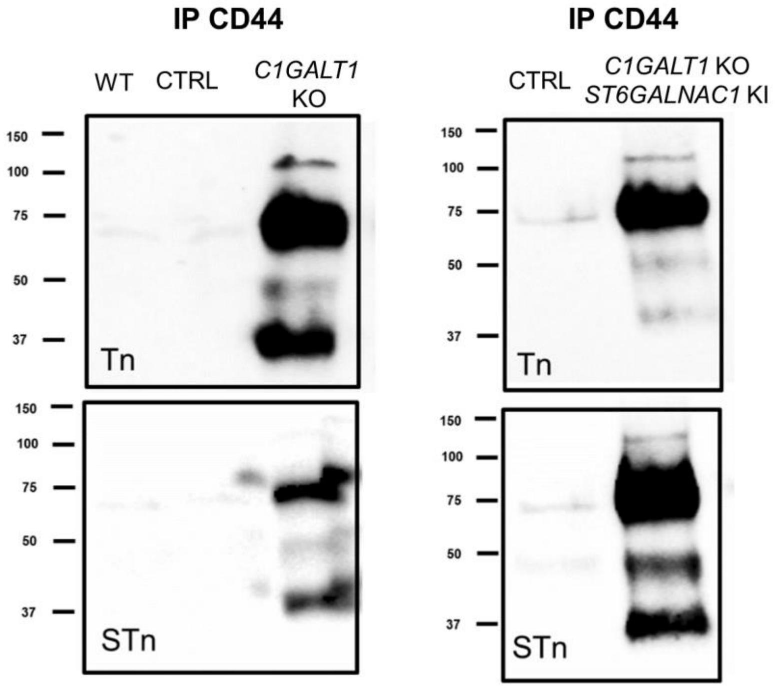

Figure S10. Western Blots show co-expression of CD44 and short-chain *O*-glycans, namely STn and Tn, in glycoengineered T24 cells. CD44 was immunoprecipitated from membrane protein extracts of T24 *C1GALT1* KO, T24 *C1GALT1* KO/*ST6GALNAC1* KI and corresponding controls and then blotted for STn, Tn, and CD44. A demarked expression of CD44-STn and CD44-Tn was detected for both cell model mainly in bands at 75 kDa and bellow.

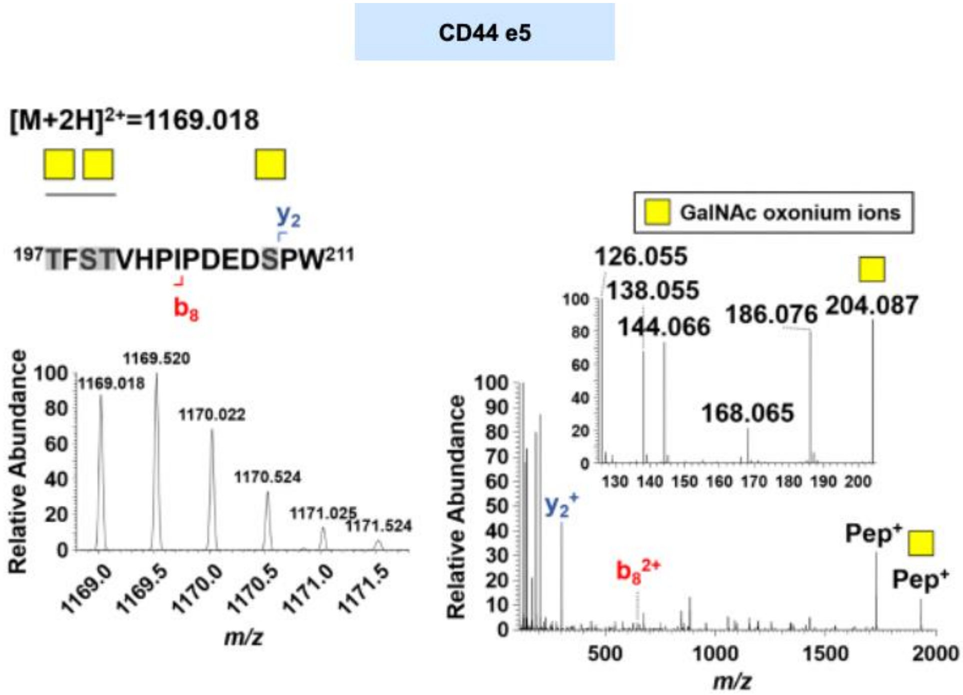

Figure S11. CD44-Tn glycopeptides belonging to the conserved extracellular domain identified in T24 *C1GALT1*KO cells by nanoLC-HCD-MS/MS. MS/MS spectrum highlights the typical HexNAC oxonium ion (*m/z* 204.087) as well as other oxonium ion fragments consistent with GalNAc (highlighted at *m/z* 138.055 and 144.066). The MS/MS spectrum of the CD44-Tn glycopeptide also highlights peptide ions with GalNAc losses. The predicted and possible glycosites are identified in grey.

Table S1. Tailored-made Taqman Gene Expression Assay ID's to specifically detect mRNA encoding for total CD44 and 4 splicing variants.

Gene	TaqMan Gene Expression Assay ID	Exon junction Boundary	Interrogated sequences
**CD44 Total**	Hs01075864_m1	e3-e4	NM_000610.3 (CD44 v2-10: ENST00000428726.8)<br>NM_001001389.1 (CD44 v3-10: ENST00000415148.6)<br>NM_001001390.1 (CD44 v8-10: ENST00000433892.6)<br>NM_001001391.1(CD44s: ENST00000263398.11)<br>NM_001202555.1 (CD44v10: ENST00000434472.6)<br>NM_001202556.1(CD44s-exon15: ENST00000352818.8)<br>NM_001202557.1 (CD44st: ENST00000442151.6)
**CD44 e5-v2**	Hs01075866_m1	e5-v6 (corresponds to exon v2 of canonical CD44)	NM_000610.3 (CD44 v2-10: ENST00000428726.8)
**CD44 e5-v3**	Hs01081480_m1	e5-v6 (corresponds to exon v3 of canonical CD44)	NM_001001389.1 (CD44 v3-10: ENST00000415148.6)
**CD44 e5-v8**	Hs01081475_m1	e5-v6 (corresponds to exon v8 of canonical CD44)	NM_001001390.1 (CD44 v8-10: ENST00000433892.6)
**CD44 e5-e15**	Hs01081473_m1	e5-v6 (corresponds to exon e15 of canonical CD44)	NM_001001391.1 (CD44s: ENST00000263398.11)<br>NM_001202557.1 (CD44st: ENST00000442151.6)

**CD44sol**	Hs01081469_m1	v2(partial)-v3(partial)	NM_001001392.1 (CD44sol: ENST00000278386.10)

Table S2. List of antibodies and lectins used in this manuscript.

Antigen	Antibody/Lectin	Source	Reference	Clone	Immunogens/targets
**CD44**	Rabbit polyclonal to CD44 antibody	Abcam	ab157107	Not provided	Synthetic peptide within Human CD44 a.a 650-750
**STn**	Mouse monoclonal to tag 72	Abcam	ab199002	B72.3+CC49	Membrane-enriched fraction of a human breast carcinoma liver metastasis [B72.3]; Purified TAG-72 protein [CC49]
**Tn**	Biotinylated and FITC-labeled VVA lectin	Vector Laboratories	B-1235-2<br>FL-1231-2	Not provided	α- or β-linked terminal N-acetylgalactosamine structures
**T and ST after desialylation with α-neuraminidase**	FITC-labeled PNA lectin	Vector Laboratories	FL-1071	Not provided	Gal (β-1,3) GalNAc structures
**B2M**	Rabbit monoclonal to beta 2 Microglobulin	Abcam	ab75853	EP2978Y	Not provided

Table S3. CD44 isoforms identified in 5637 and T24 cells by RNAseq.

Ensembl	Uniprot	CD44 Isoforms
ENST00000263398.11	P16070-12	**CD44s**
ENST00000526025.2	E9PKC6	**CD44splice E2**
ENST00000415148.6	P16070-4	**CD44v3-10**
ENST00000428726.8	P16070-1	**CD44v2-10**
ENST00000433892.6	P16070-10	**CD44v8-10**
ENST00000278386.10	P16070-19	**CD44sol**
ENST00000434472.6	P16070-11	**CD44v10**
ENST00000352818.8	P16070-18	**CD44s-exon15**
ENST00000442151.6	P16070-15 or H0Y5E4	**CD44st**
ENST00000526669.6	H0YD13	-
ENST00000425428.6	Q86UZ1	-
ENST00000528086.5	-	-
ENST00000526000.6	H0YDW7	-
ENST00000279452.10	H0Y2P0	-
ENST00000531118.5	-	-
ENST00000528455.5	H0YD17	-
ENST00000531873.5	H0YD90	-
ENST00000525209.5	-	-

Table S4. Glycopeptides identified by nanoLC-HCD/CID-MS/MS in glycoproteogenomics settings for 5637 cells.

Peptide sequences	5637								
	Glycan chains								
	HexNAc	HexNAcNeuAc	HexHexNAc	HexHexNAcNeuAc	HexHexNAcNeuAc(2)	HexNAc(2)	HexNAc(2)Hex	HexNAc(2)Hex(2)NeuAc	HexNAc(1)Hex(1)Fuc
**CD44v2-10**									
LFLPSESKNHLHTTTQMAGTSsNtISAGW			x				x		
lPsEsKNHLHTTTQMAGTSSNTISAGW	x		x						
LFLPSESKNHLHtTTQMAGTsSnTIsAGW	x		x						
LFLPSESKNHLHtttQMAGTSSnTISAGW			x						
DWFsW									x
FsWLF									x
LFLPSESKNHLHTTtQMAGTSSNTISAGW					x				
dWFsW					x				
FsWLF						x			
LFLPSESKnHLHTTTQmAGtSSnTISAGW	x								
fsWLF								x	
FsWLF							x		
LFLPSESKNHLHtTTQmAGtSSNTIsAGW	x					x			
DWFsW			x						
LFLPsEsKNHLHTTTQmAGTSSNTISAGW						x			
LPsEsKNHLHTTTQMAGTSSNTISAGW									
LPSESKNHLHtTTQMAGTSSNTIsAGW									
LFLPsESKNHLHTTTQmAGTSSNTISAGW									
LFLPSESKnHLHtttQMAGTSSNTISAGW									
LPSESKnHLHtttQMAGTSSNTISAGW									
LPSESKnHLHTttQMAGTSSNTISAGW									
LPSESKNHLHTtTQmAGTSSNTISAGW									
LFLPsESKNHLHTTtQMAGTSSNTISAGW									
LFLPSEsKNHLHTTTQmAGTsSNTISAGW									
LFLPsEsKNHLHTTTQmAGTSSNTISAGW									
LPSESKNHLHTtTQmAGTSSNTISAGW									
sWLF									
sWLF									
FsWLF									
LFLPsESKnHLHTtTQMAGTSSNTISAGW									
STVHPIPDEDSPWITDStDRIPAtRDQDtF									
dWFsW		x							
sWLF									
FsWLF									
DWFsW									
FsWLF									
DWFsW									
**CD44v3-10**									
ItDSTDRIPATSTSSnTISAGW									
ItDSTDRIPAtStSSNTISAGW									
ITDSTDRIPATStSSNTISAGW									
ITDSTDRIPATSTSsNTIsAGW		x							x
ITDStDRIPATSTssNTIsAGW	x		x			x			
ITDStDRIPATSTsSNTIsAGW			x			x	x		
iTDSTDRIPATSTsSNTISAGW			x						
iTDSTDRIPATSTSsnTISAGW	x								
ITDSTDRIPAtStSSnTIsAGW	x		x						
ITDSTDRIPATSTssnTIsAGW	x					x			
ITDSTDRIPATSTssnTIsAGW									
ITDSTDRIPATSTssnTIsAGW									
ITDSTDRIPATSTssnTIsAGW									
**CD44s**									
StVHPIPDEDsPWITDSTDRIPATRDQDtF	x		x				x		
stVHPIPDEDsPWITDsTDRIPATRDQDTF	x		x						
ITDStDRIPATRDQDTF									x
sTVHPIPDEDSPWITDSTDRIPAtRDQDtF									
STVHPIPDEDSPWItDsTDRIPATRDQDTF									
StVHPIPDEDSPWItDSTDRIPAtRDQDTF									
ItDsTDRIPATRDQDTF									
sTVHPIPDEDsPWITDsTDRIPATRDQDTF									
ITDstDRIPAtRDQDtF									
ItDStDRIPAtRDQDTF									
STVHPIPDEDSPWItDStDRIPAtRDQDTF									
sTVHPIPDEDSPWItDStDRIPATRDQDTF									
ItDStDRIPAtRDQDTF									
ITDStDRIpAtRDQDtF									
STVhPIPDEDSpWITDsTDRIPATRDQDTf									
STVHPIPDEDsPWITDstDRIpATRDQDTF									
**CD44sol**									
qWScGGQKAKWtQRRGQQVSGnGAF						x			
GEQGVVRNsRPVY						x			
GEQGVVRNsRPVY	x								
GEQGVVRnsRPVYDs	x		x						
QWscGGQKAKW	x								
tQRRGQQVsGNGAF			x						
TQRRGQQVsGnGAF	x								
etcsLHcSQQSKKVWAEEKASDQQW						x	x		
scGGQKAKWTQRRGQQVsGnGAF	x								
ETcSLHcsQQsKKVW						x			
tQRRGQQVSGNGAFGEQGVVRNsRPVYDs						x	x		
TQRRGQQVsGNGAF		x							
gEQGVVRNsRPVY		x							
scGGQKAKWTQRRGQQVSGNGAF									x
TQRRGQQVsGNGAF								x	
EtcSLHcSQQSKKVW									
AEEKAsDQQWQW									
ETcsLHcSQQSKKVWAEEKAsDQQWQW									
qWScGGQKAKWTQRRGQQVsGNGAF									
QWScGGQKAKWTQRRGQQVsGNGAF									

Table S5. Glycopeptides identified by nanoLC-HCD/CID-MS/MS in glycoproteogenomics settings for T24 cell line.

Peptide sequence	T24								
	Glycan chains								
	HexNAc	HexNAcNeuAc	HexHexNAc	HexHexNAcNeuAc	HexHexNAcNeuAc(2)	HexNAc(2)	HexNAc(2)Hex	HexNAc(2)Hex(2)NeuAc	HexNAc(1)Hex(1)Fuc
CD44v2-10									
LFLPSESKNHLHTTTQMAGTSsNtISAGW			x				x		
IPsEsKNHLHTTTQMAGTSSNTISAGW	x		x						
LFLPSESKNHLHtTTQMAGTsSnTIsAGW									
LFLPSESKNHLHtttQMAGTSSnTISAGW									
DWFsW									
FsWLF									x
LFLPSESKNHLHTTtQMAGTSSNTISAGW					x				
dWFsW									
FsWLF						x			
LFLPSESKnHLHTTTQmAGtSSnTISAGW									
fsWLF								x	
FsWLF							x		
LFLPSESKNHLHtTTQmAGtSSNTIsAGW									
DWFsW									
LFLPsEsKNHLHTTTQmAGTSSNTISAGW						x			
LPsEsKNHLHTTTQMAGTSSNTISAGW									
LPSESKNHLHtTTQMAGTSSNTIsAGW					x				x
LFLPsESKNHLHTTTQmAGTSSNTISAGW		x							
LFLPSESKnHLHtttQMAGTSSNTISAGW						x	x		
LPSESKnHLHtttQMAGTSSNTISAGW	x		x				x		
LPSESKnHLHTttQMAGTSSNTISAGW							x		
LPSESKNHLHTtTQmAGTSSNTISAGW									x
LFLPsESKNHLHTTtQMAGTSSNTISAGW		x							x
LFLPSEsKNHLHTTTQmAGTsSNTISAGW			x						
LFLPsEsKNHLHTTTQmAGTSSNTISAGW	x								
LPSESKNHLHTtTQmAGTSSNTISAGW		x							
sWLF									x
sWLF								x	
FsWLF				x					
LFLPsESKnHLHTtTQMAGTSSNTISAGW									
STVHPIPDEDSPWITDStDRIPAtRDQDtF									
dWFsW									
sWLF									
FsWLF									
DWFsW									
FsWLF									
DWFsW									
CD44v3-10									
ItDSTDRIPATSTSSnTISAGW						x			
ItDSTDRIPAtStSSNTISAGW	x						x		
ITDSTDRIPATStSSNTISAGW			x						
ITDSTDRIPATSTSsNTIsAGW									
ITDStDRIPATSTssNTIsAGW									
ITDStDRIPATSTsSNTIsAGW									
iTDSTDRIPATSTsSNTISAGW									
iTDSTDRIPATSTSsnTISAGW									
ITDSTDRIPAtStSSnTIsAGW									
ITDSTDRIPATSTssnTIsAGW									
ITDSTDRIPATSTssnTIsAGW									
ITDSTDRIPATSTssnTIsAGW									
ITDSTDRIPATSTssnTIsAGW									
CD44s									
StVHPIPDEDsPWITDSTDRIPATRDQDtF	x		x				x		
stVHPIPDEDsPWITDsTDRIPATRDQDTF	x		x						
ITDStDRIPATRDQDTF									x
sTVHPIPDEDSPWITDSTDRIPAtRDQDtF			x				x		
STVHPIPDEDSPWItDsTDRIPATRDQDTF		x						x	
StVHPIPDEDSPWItDSTDRIPAtRDQDTF	x		x				x		
ItDsTDRIPATRDQDTF						x			
sTVHPIPDEDsPWITDsTDRIPATRDQDTF		x							x
ITDstDRIPAtRDQDtF		x			x				x
ItDStDRIPAtRDQDTF									
STVHPIPDEDSPWItDStDRIPAtRDQDTF									
sTVHPIPDEDSPWItDStDRIPATRDQDTF	x					x			
ItDStDRIPAtRDQDTF									
ITDStDRIpAtRDQDtF									
STVhPIPDEDSpWITDsTDRIPATRDQDTf									
STVHPIPDEDsPWITDstDRIpATRDQDTF									
CD44sol									
qWScGGQKAKWtQRRGQQVSGnGAF									
GEQGVVRNsRPVY									
GEQGVVRNsRPVY	x								
GEQGVVRnsRPVYDs									
QWscGGQKAKW									
tQRRGQQVsGNGAF									
TQRRGQQVsGnGAF									
etcsLHcSQQSKKVVWAEEKASDQQW						x	x		
scGGQKAKWTQRRGQQVsGnGAF	x								
ETcSLHcsQQsKKVW									
tQRRGQQVSGNGAFGEQGVVRNsRPVYDs									
TQRRGQQVsGNGAF		x							
gEQGVVRNsRPVY		x							
scGGQKAKWTQRRGQQVSGNGAF									x
TQRRGQQVsGNGAF								x	
EtcSLHcSQQSKKVW				x					
AEEKAsDQQWQW		x							
ETcsLHcSQQSKKVWAEEKAsDQQWQW			x			x			
qWScGGQKAKWTQRRGQQVsGNGAF									x
QWScGGQKAKWTQRRGQQVsGNGAF		x							

Table S6. Glycopeptides identified by nanoLC-HCD/CID-MS/MS in glycoproteogenomics settings for CD44s^{high} MIBC showing areas of CD44 and STn co-localization.

Peptide sequence	MIBC	
	Glycan chains	
	HexNAc	HexNAcNeuAc
**CD44v2-10**		
LFLPSESKNHLHTTTQMAGTSsNtISAGW		
IPsEsKNHLHTTTQMAGTSSNTISAGW		
LFLPSESKNHLHtTTQMAGTsSnTIsAGW		
LFLPSESKNHLHtttQMAGTSSnTISAGW		
DWFsW		
FsWLF		
LFLPSESKNHLHTTtQMAGTSSNTISAGW		
dWFsW		
FsWLF		
LFLPSESKnHLHTTTQmAGtSSnTISAGW		
fsWLF		
FsWLF		
LFLPSESKNHLHtTTQmAGtSSNTIsAGW		
DWFsW		
LFLPsEsKNHLHTTTQmAGTSSNTISAGW		
LPsEsKNHLHTTTQMAGTSSNTISAGW		
LPSESKNHLHtTTQMAGTSSNTIsAGW		
LFLPsESKNHLHTTTQmAGTSSNTISAGW		
LFLPSESKnHLHtttQMAGTSSNTISAGW		
LPSESKnHLHtttQMAGTSSNTISAGW		
LPSESKnHLHTttQMAGTSSNTISAGW		
LPSESKNHLHTtTQmAGTSSNTISAGW		
LFLPsESKNHLHTTtQMAGTSSNTISAGW		
LFLPSEsKNHLHTTTQmAGTsSNTISAGW		
LFLPsEsKNHLHTTTQmAGTSSNTISAGW	x	
LPSESKNHLHTtTQmAGTSSNTISAGW	x	
sWLF		
sWLF		
FsWLF		
LFLPsESKnHLHTtTQMAGTSSNTISAGW	x	x
STVHPIPDEDSPWITDStDRIPAtRDQDtF		
dWFsW		
sWLF		x
FsWLF	x	
DWFsW	x	
FsWLF		x
DWFsW		x
**CD44v3-10**		
ItDSTDRIPATSTSSnTISAGW		
ItDSTDRIPAtStSSNTISAGW		
ITDSTDRIPATStSSNTISAGW		
ITDSTDRIPATSTSsNTIsAGW		
ITDStDRIPATSTssNTIsAGW		
ITDStDRIPATSTsSNTIsAGW		
iTDSTDRIPATSTsSNTISAGW		
iTDSTDRIPATSTSsnTISAGW		
ITDSTDRIPAtStSSnTIsAGW		
ITDSTDRIPATSTssnTIsAGW		
ITDSTDRIPATSTssnTIsAGW	x	x
ITDSTDRIPATSTssnTIsAGW		x
ITDSTDRIPATSTssnTIsAGW	x	
**CD44s**		
StVHPIPDEDsPWITDSTDRIPATRDQDtF		
stVHPIPDEDsPWITDsTDRIPATRDQDTF		
ITDStDRIPATRDQDTF		
sTVHPIPDEDSPWITDSTDRIPAtRDQDtF		
STVHPIPDEDSPWItDsTDRIPATRDQDTF		
StVHPIPDEDSPWItDSTDRIPAtRDQDTF		
ItDsTDRIPATRDQDTF		
sTVHPIPDEDsPWITDsTDRIPATRDQDTF		
ITDstDRIPAtRDQDtF		
ItDStDRIPAtRDQDTF	x	
STVHPIPDEDSPWItDStDRIPAtRDQDTF	x	
sTVHPIPDEDSPWItDStDRIPATRDQDTF		
ItDStDRIPAtRDQDTF		x
ITDStDRIpAtRDQDtF	x	x
STVhPIPDEDSpWITDsTDRIPATRDQDTf		x
STVHPIPDEDsPWITDstDRIpATRDQDTF	x	x
**CD44sol**		
qWScGGQKAKWtQRRGQQVSGnGAF		
GEQGVVRNsRPVY		
GEQGVVRNsRPVY	x	
GEQGVVRnsRPVYDs		
QWscGGQKAKW		
tQRRGQQVsGNGAF		
TQRRGQQVsGnGAF		
etcsLHcSQQSKKVWAEEKASDQQW		
scGGQKAKWTQRRGQQVsGnGAF	x	
ETcSLHcsQQsKKVW		
tQRRGQQVSGNGAFGEQGVVRNsRPVYDs		
TQRRGQQVsGNGAF		
gEQGVVRNsRPVY		x
scGGQKAKWTQRRGQQVSGNGAF		
TQRRGQQVsGNGAF		
EtcSLHcSQQSKKVW		
AEEKAsDQQWQW		
ETcsLHcSQQSKKVWAEEKAsDQQWQW		
qWScGGQKAKWTQRRGQQVsGNGAF		
QWScGGQKAKWTQRRGQQVsGNGAF		x

Table S7. Glycopeptides identified by nanoLC-HCD/CID-MS/MS in glycoproteogenomics settings for T24 *C1GALT1* KO cell model.

Peptide sequence	T24 *C1GALT1* KO		
	Glycan chains		
	HexNAc	HexNAcNeuAc	HexNAc(2)
**CD44v2-10**			
LFLPSESKNHLHTTTQMAGTSsNtISAGW			
IPsEsKNHLHTTTQMAGTSSNTISAGW			
LFLPSESKNHLHtTTQMAGTsSnTIsAGW			
LFLPSESKNHLHtttQMAGTSSnTISAGW			
DWFsW			
FsWLF			
LFLPSESKNHLHTTtQMAGTSSNTISAGW			
dWFsW			
FsWLF			
LFLPSESKnHLHTTTQmAGtSSnTISAGW			
fsWLF			
FsWLF			
LFLPSESKNHLHtTTQmAGtSSNTIsAGW			
DWFsW			
LFLPsEsKNHLHTTTQmAGTSSNTISAGW			
LPsEsKNHLHTTTQMAGTSSNTISAGW			
LPSESKNHLHtTTQMAGTSSNTIsAGW			
LFLPsESKNHLHTTTQmAGTSSNTISAGW			
LFLPSESKnHLHtttQMAGTSSNTISAGW			
LPSESKnHLHtttQMAGTSSNTISAGW			
LPSESKnHLHTttQMAGTSSNTISAGW			
LPSESKNHLHTtTQmAGTSSNTISAGW			
LFLPsESKNHLHTTtQMAGTSSNTISAGW			
LFLPSEsKNHLHTTTQmAGTsSNTISAGW			
LFLPsEsKNHLHTTTQmAGTSSNTISAGW	x		
LPSESKNHLHTtTQmAGTSSNTISAGW			
sWLF			
sWLF			
FsWLF			
LFLPsESKnHLHTtTQMAGTSSNTISAGW	x	x	
STVHPIPDEDSPWITDStDRIPAtRDQDtF	x		
dWFsW			
sWLF			
FsWLF			
DWFsW			
FsWLF			
DWFsW			
**CD44v3-10**			
ItDSTDRIPATSTSSnTISAGW			
ItDSTDRIPAtStSSNTISAGW			
ITDSTDRIPATStSSNTISAGW			
ITDSTDRIPATSTSsNTIsAGW			
ITDStDRIPATSTssNTIsAGW			
ITDStDRIPATSTsSNTIsAGW			
iTDSTDRIPATSTsSNTISAGW			
iTDSTDRIPATSTSsnTISAGW			
ITDSTDRIPAtStSSnTIsAGW			
ITDSTDRIPATSTssnTIsAGW			
ITDSTDRIPATSTssnTIsAGW			
ITDSTDRIPATSTssnTIsAGW			
ITDSTDRIPATSTssnTIsAGW			
**CD44s**			
StVHPIPDEDsPWITDSTDRIPATRDQDtF			
stVHPIPDEDsPWITDsTDRIPATRDQDTF			
ITDStDRIPATRDQDTF			
sTVHPIPDEDSPWITDSTDRIPAtRDQDtF			
STVHPIPDEDSPWItDsTDRIPATRDQDTF			
StVHPIPDEDSPWItDSTDRIPAtRDQDTF			
ItDsTDRIPATRDQDTF			
sTVHPIPDEDsPWITDsTDRIPATRDQDTF			
ITDstDRIPAtRDQDtF			
ItDStDRIPAtRDQDTF	x		
STVHPIPDEDSPWItDStDRIPAtRDQDTF	x		
sTVHPIPDEDSPWItDStDRIPATRDQDTF			
ItDStDRIPAtRDQDTF			
ITDStDRIpAtRDQDtF			
STVhPIPDEDSpWITDsTDRIPATRDQDTf			
STVHPIPDEDsPWITDstDRIpATRDQDTF			
**CD44sol**			
qWScGGQKAKWtQRRGQQVSGnGAF			
GEQGVVRNsRPVY			
GEQGVVRNsRPVY			
GEQGVVRnsRPVYDs			
QWscGGQKAKW			
tQRRGQQVsGNGAF			
TQRRGQQVsGnGAF	x		
etcsLHcSQQSKKVWAEEKASDQQW			
scGGQKAKWTQRRGQQVsGnGAF	x		
ETcSLHcsQQsKKVW			
tQRRGQQVSGNGAFGEQGVVRNsRPVYDs			
TQRRGQQVsGNGAF		x	
gEQGVVRNsRPVY			
scGGQKAKWTQRRGQQVSGNGAF			
TQRRGQQVsGNGAF			
EtcSLHcSQQSKKVW			
AEEKAsDQQWQW			
ETcsLHcSQQSKKVWAEEKAsDQQWQW			
qWScGGQKAKWTQRRGQQVsGNGAF			
QWScGGQKAKWTQRRGQQVsGNGAF			
